# Effect of radiation-induced vacancy saturation on the first-order phase transformation in nanoparticles: insights from a model

**DOI:** 10.3762/bjnano.15.117

**Published:** 2024-11-21

**Authors:** Aram Shirinyan, Yuriy Bilogorodskyy

**Affiliations:** 1 “Laboratory of composite materials for nuclear-hydrogen energy”, Department of nuclear-physical research, Institute of Applied Physics of National Academy of Sciences of Ukraine (Sumy), room 606, building 3, Nauki avenue, 46, 03028, Kyiv - 28, Ukraine

**Keywords:** α-phase, β-phase, chemical rate theory, Fe, nanoparticle, nucleation, phase stability diagram, polymorphic phase transision, radiation stability, thermodynamics, vacancy saturation

## Abstract

By employing a model of nanomaterials with polymorphic phase transitions and using a thermodynamic approach to describe the effects of vacancy saturation, irradiation dose, powder dispersion, and surface energies, we demonstrate the possibility of radiation-induced phase transitions and the zones of radiation stability for nanoparticles. We utilize nanoparticles exhibiting transitions from the body-centered cubic α phase to the face-centered cubic β phase, and the reverse transition from β phase to α phase, as a model system for first-order phase transformations. We incorporate nucleation through the appearance and growth of the nucleus of a new phase, resulting in the formation of a two-phase α+β system, and we highlight the importance of accounting for nucleation. Our model study reveals that very small α-phase particles are unstable (while very small β-phase particles are stable) because of surface effects. There is an intermediate zone of sizes and parameters where radiation-induced defects become important so that the α-phase particle is unstable without irradiation but becomes stable under irradiation. For large sizes and low temperatures, the α→β transformation cannot occur regardless of irradiation because of bulk driving forces; initially, α-phase particles are stable, whereas the β-phase particles are unstable. In some cases, nucleation requires a large additional energy change, resulting in a low probability of phase change fluctuations. This behavior is confirmed by calculations for iron particles under irradiation. Substances characterized by high vacancy migration energy, small diffusion coefficients of defects, and low temperatures of first-order phase transitions can serve as suitable candidates for radiation-induced phase transitions in nanosystems. Ceramic nanomaterials, which possess high vacancy migration energy, will have their behavior significantly influenced by radiation doses. In contrast, most metals exhibit small vacancy migration energy and demonstrate better resistance to irradiation, making them recommended candidates for nuclear materials.

## Introduction

Solid metal or ceramic nanoparticles with a diameter in the range of 1–100 nm, when placed in an inert environment, can be considered highly dispersed composite materials (HDCMs). HDCMs exhibit potential for the use under conditions of high temperatures and radiation exposure, making them promising materials for new-generation modular nuclear reactors, advanced charge storage applications, and other emerging nanotechnologies. When HDCMs are exposed to radiation, such as ion bombardment or exposure to high-energy radiation sources, defects (vacancies, interstitials, point defect clusters, voids, and interstitial loops) are created in the crystal lattice because of the displacement of atoms. These defects can significantly alter the structural, mechanical, and electronic properties of materials. This prompts the questions: How do radiation-induced defects influence first-order phase transformations in nanoscale systems? Can radiation-induced defects initiate polymorphic transitions or amorphization in metallic/ceramic nanoparticles, leading to changes in the crystal structure? Is it feasible to establish a fundamental basis to explain the behavior of materials under irradiation?

Most nuclear materials have not been tested beyond an irradiation dose of 200 displacements per atom (dpa) (equivalent to 40 years of service). Under irradiation, the main point defects are vacancies and interstitials. Point defects can develop into clusters of dislocations, stacking faults, or voids. They can also relax onto existing sinks such as dislocation loops, grain boundaries, phase interfaces, and cavities [1–2]. Experimental studies on Pd have shown that the defect density generally increases with grain size; in grains smaller than 30 nm, no defects were observed [3], suggesting that large defects (clusters and dislocations) do not exist in small nanoparticles.

One possible explanation is based on the fact that the movement of dislocations is impeded by particle surfaces (grain boundaries) quite rapidly. For example, a transmission electron microscopy study (irradiation with Kr ions at 1 MeV at room temperature and an average defect generation rate of about 2 × 10^−3^ dpa·s^−1^) showed that, in nanosilver, a dislocation loop migrates to the free surface of the particle within 0.1 s [4]. This suggests that dislocation loops and interstitials are leveled out fairly quickly in nanoparticles, making vacancies the main defects that affect the material’s properties.

According to experimental data, under operating conditions, HDCMs are susceptible to risks such as radiation-induced vacancy saturation (the accumulation of vacancy-type defects), swelling (an increase in linear dimensions and volume upon irradiation), damage, amorphization (crystal-to-glass transition), and other phase transformations (phase-to-phase transitions) [5–8]. Despite the interest in these materials, the behavior of nanoparticles under irradiation and their peculiarities are not yet completely understood. One of the important issues is the influence of vacancy saturation on phase changes and phase stability.

A literature review reveals that the stability of materials under irradiation is influenced by numerous factors. Some of these characteristic factors include the elemental composition and chemical structure, the microstructure of the material (including grain boundaries, defects, dislocations, and interfaces), the dose and energy of the radiation source, different types of radiation, environmental conditions, the purity and homogeneity of the material, and the crystal structure and phase stability. Let us briefly consider these publications and highlight characteristic factors to facilitate understanding and subsequent description.

It is important to distinguish between two types of point defects, that is, (i) thermal-equilibrium defects (vacancies and interstitials that exist without irradiation treatment) and (ii) radiation-induced defects. Point defects caused by radiation are formed when a fast moving ion knocks an atom from its initial lattice position. The appearance of point defects increases the energy of the crystal, as energy is required to create each defect. For example, the energy of vacancy formation in the face-centered cubic (fcc) Cu lattice is about 1 eV, and in the body-centered cubic (bcc) Fe lattice it is about 1.5–2.0 eV; the energy of interstitial formation ranges from 2 to 4 eV. It is accepted that interstitials are mobile at room (low) temperature because of significantly less migration energies of 0.01–0.50 eV, whereas vacancies are mobile at very high temperatures. Disorder can arise from the recombination of these defects [1–9]. In metals, for instance, the equilibrium concentration of thermal vacancies, even at high pre-melting temperatures, reaches values of only about 0.1% [10–11]. Therefore, in the following, we will focus on radiation-induced vacancies, assuming that the concentration of radiation-induced point defects at characteristic temperatures (far from melting) exceeds the concentration of thermal-equilibrium defects.

The behavior of HDCMs under irradiation highly depends on their size. For example, when TiN nanograins are irradiated with He^+^ ions, their amorphization leads to a reduction in nanohardness, and this reduction is strongly correlated with the grain size [12]. Phase instability (radiation-induced amorphization) is observed in zirconia nanoparticles (ZrO_2_) embedded in nanocrystalline composites. ZrO_2_ nanoparticles can be amorphized at an irradiation dose of 0.9 dpa, whereas bulk zirconia remains stable with respect to amorphization up to 680 dpa [5]. Additionally, experimental data indicate that materials containing coherent-to-the-matrix dispersed particles may experience reduced swelling under irradiation compared to similar materials lacking such precipitates. For example, nanocrystalline solids of ZrO_2_ or Pd demonstrate high resistance to radiation-induced defect production compared to coarse-grained polycrystals with the same chemical composition [3]. Consequently, a significant increase in radiation tolerance is anticipated in nanocrystalline materials compared to bulk solids with conventional grain sizes. Therefore, it is expected that interfaces such as coherent and incoherent boundaries in HDCMs will act as sinks to promote point defect annihilation [6].

In many cases, multicomponent alloys and HDCMs demonstrate greater stability compared to nanocrystalline pure materials. For example, nanocrystalline NiFe alloys can withstand a much higher radiation dose, twice that of nanocrystalline Ni, and are more stable under prolonged irradiation compared to nanocrystalline elemental Ni [7]. Additionally, high radiation tolerance was observed in crystalline Fe/amorphous SiOC nanolaminates [8].

Long-term irradiation treatments have revealed phase transformations in HDCMs, such as the crystallization of an earlier formed amorphous state (re-crystallization) or a change in the basic crystalline state. For example, a two-phase TiCr alloy undergoes phase transformation when irradiated with Kr^+^ ions with an energy of 1 MeV [13]. In another study, a bcc→fcc phase transition was observed in an α-FeNi alloy due to radiation of self-ions at 673 K and a dose of approximately 1.2 dpa [14]. Furthermore, changes in the structural properties of Ni nanostructures due to ion bombardment have been reported in [15]. Interestingly, certain types of radiation ions have shown a positive effect on the crystal structure, leading to an increase in the degree of crystallinity after the austenitic annealing of defects. However, in cases where the structure is rebuilt, the role of vacancies becomes less obvious. This suggests that other factors must be considered to better understand the transformations in HDCMs under irradiation.

A recent study reported a phase transformation in a 25 nm thick nanocrystalline Au thin film through in situ ion irradiation, observed using atomic-resolution transmission electron microscopy [16]. The gold sample was irradiated with 2.8 MeV Au^4+^ ions at 200 °C with a fluence of approximately 10^14^ ions·cm^−2^ (equivalent to a dose of 10 dpa). A combination of surface- and radiation-induced effects led to a polymorphic phase change, transforming the high-density fcc structure to a low-density hexagonal close-packed crystallographic phase.

The investigation of the radiation stability of nanocrystalline single-phase multicomponent alloys (NiFe, NiCoFe, and NiCoCr) using molecular dynamics simulations reveals that the critical irradiation dose for nanocrystallinity collapse varies among different simulation cells. Not only the size, but also the crystallographic orientation, shape of the grains, and structure of the grain boundaries have a strong impact on the stability of the nanocrystalline phase [7]. In all cells, the grains undergo a phase transition from a pure high-density fcc phase to a mixture of fcc and bcc phases during prolonged irradiation. These simulations confirm that the phase transition occurs because of the ground-state energies of the compositions rather than the irradiation itself. Consequently, it would be intriguing to identify other systems capable of undergoing, for example, polymorphic transformations or amorphization under irradiation, transitioning from a high-density phase to a low-density phase. One such example will be discussed further.

There is a general deficiency in theoretical descriptions, particularly regarding thermodynamic calculations, which could elucidate the phase stability and radiation stability of nanodispersed particles under irradiation. In essence, there is a lack of robust theory to inform studies of HDCMs under irradiation. The authors have identified only a few papers proposing a thermodynamic assessment [17–20]. Our aim in this work is to fill this gap.

Shen’s proposed qualitative framework suggests that the grain size of a material influences its resistance to amorphization and the removal of radiation defects by altering the Gibbs free energy and kinetic rate theory [17]. Shen delineates five size-dependent regions that govern the material’s response to irradiation. Nevertheless, Shen’s approach remains qualitative, highlighting the need for a more comprehensive thermodynamic assessment to enhance our understanding of HDCMs’ behavior under irradiation.

Recently, we attempted to adapt Shen’s model for polymorphic transformations in nanoscale Fe systems (conference report, providing an initial approximation to the formulation of the problem) [19]. However, certain assumptions raise doubts about the results, namely, (i) the absence of vacancies in the secondary phase, which should be released under irradiation, (ii) the consideration of vacancy diffusion coefficients as constants, rather than exponentially dependent on migration energy and temperature, and (iii) the modeling of thermodynamic parameters, such as the enthalpy and entropy of vacancy formation in Fe.

First-order phase transformations are accompanied by nucleation and the overcoming of energy barriers. To our knowledge, the consideration of nucleation energy barriers and the alteration of surface energies during transformation under irradiation has been largely overlooked. This raises questions about the potential outcomes when the surface energy decreases because of the emergence of a new phase. Other factors contributing to non-uniform behavior and strongly influencing transformation patterns may also warrant investigation. In this regard, solid nanomaterials with phase change and reduction in surface tension serve as suitable systems for elucidation and comparison.

In summary, there is a competition among various energetic factors influencing phase stability and transformations in HDCMs during irradiation. These factors include (i) the bulk thermodynamic stimulus for phase change, (ii) the contribution of surface energy due to a high percentage of surface atoms, (iii) interfaces acting as sinks for radiation-induced point defects, (iv) the accumulation of defects (saturation of vacancies) in the material as a driving force of phase changes, and (v) the nucleation of a new phase. We leverage this competition to develop a fundamental description, employing both a thermodynamic approach based on the calculation of Gibbs free energy and a kinetic approach based on chemical rate theory. Given the complexity arising from multiple factors, it is evident that a simple theoretical description may not suffice. Therefore, our aim is to tackle the problem comprehensively and emphasize the most significant aspects of phase stability under irradiation.

To the best of our knowledge, this is the first work that simultaneously takes into account the above factors in a comprehensive thermodynamic approximation. As our model system, we selected a spherical nanoscale particle in an inert medium, for which we utilized the parameters necessary for calculations. Our aim is to investigate the effects of powder dispersion, surface energies of phases, and vacancy saturation on the radiation stability and first-order phase changes of spherical nanoparticles. Specifically, our objective in this work is to study the phase transitions from the bcc α phase to the fcc β phase under irradiation, as well as the reverse transition from β phase to α phase under irradiation. It is worth noting that the thermodynamic analysis and conclusions drawn from this study are applicable for understanding amorphization and polymorphic phase transitions in both metals and ceramics. In the present study we investigate model systems with structure change, but without composition change, in order to study the effect of radiation on structure change.

The paper is organized as follows: In Section “Theory”, we develop a thermodynamic approach and discuss a kinetic model of steady-state concentrations of radiation-induced defects based on chemical rate theory. Section “Results” focuses on the model of α→β phase transition and the reverse β→α phase transition from a thermodynamics perspective. Finally, section “Discussion” discusses the effects of size and irradiation, justifying their relevance to metals and ceramics, and presents the particular case of Fe nanoparticles.

## Theory

**Figure 1:**
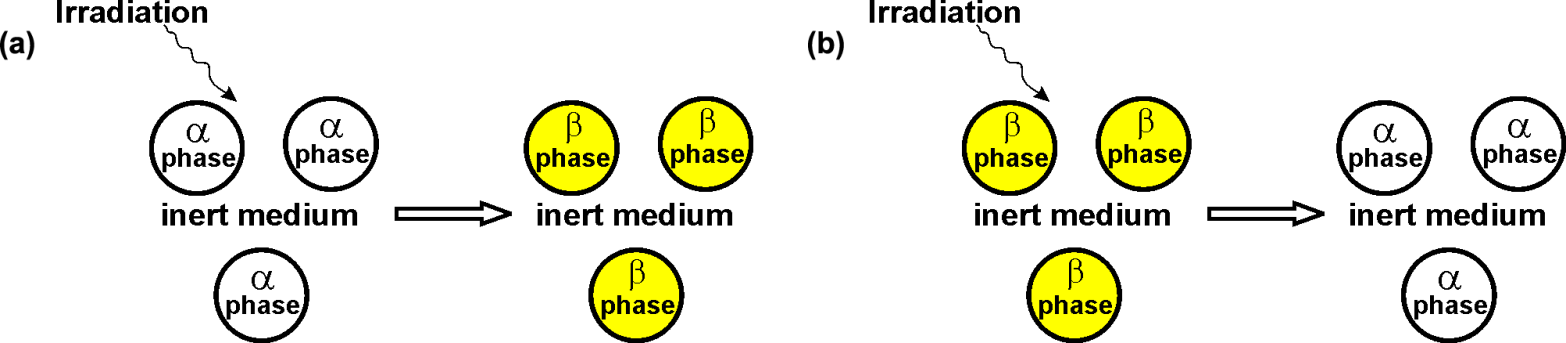
Model of a HDCM under irradiation providing a schematic representation of the irradiation treatment and first-order phase transformation of a nanopowder in an inert medium. (a) Transition from α phase to β phase and (b) transition from β phase to α phase. The solid nanoparticles are represented as identical spherical balls with a diameter *d* = 2*R*.

In this study, we define the size of a particle as its diameter in a spherical shape or the number of atoms (*N*_0_) in the nanoparticle with a given radius *R*, as depicted in Figure 1. The phase stability is evaluated based on various competing energy factors, including the degree of radiation-induced vacancy saturation, particle size, temperature, bulk energy change of the phase transition, and surface energies of phases. We define radiation stability as the resistance to phase transition, and our investigation aims to identify the factors influencing this stability. Figure 1 presents a model of a HDCM under irradiation, illustrating the irradiation treatment and the first-order phase transformations of a nanopowder within an inert medium.

Beginning with an initially homogeneous nanoscale droplet of the α phase (or the β phase) at a specific temperature, the thermodynamic analysis considers the effects of irradiation, which generate vacancies and interstitial atoms in both the interior and on the surfaces of the nanoparticle. Our approach utilizes thermodynamic calculations to determine the Gibbs free energy of a nanoparticle in various phase states with vacancy-type defects. Additionally, we consider the size-dependence of radiation-induced concentrations of point defects in our analysis. Let us proceed to discuss the thermodynamic calculations.

### Energy change without irradiation

To start, we calculate the energy change without considering the effects of irradiation. The change in Gibbs free energy of the particle, which is represented by the bulk driving force for the phase transition and the surface energy term, can be expressed as follows:


[1] $$\Delta G_{\text{p}}=\Delta G_{\text{bulk}}+\Delta G_{\text{surf}}.$$

Δ*G*_bulk_ represents the bulk Gibbs free energy change, which serves as the bulk thermodynamic stimulus for the phase transition from one phase to another. Δ*G*_surf_ denotes the surface energy change during the phase transition. Let *N*_0_ be the number of atoms in the nanoparticle with radius *R*. Then, the Gibbs free energy of an α-phase particle, *G*_α_, is:


[2] $$\Delta G_{\text{p}}=\Delta G_{\text{bulk}}+\Delta G_{\text{surf}}.$$

The total Gibbs free energy, *G*_β_, of a β-phase particle is given by:


[3] $$G_{\beta }=N_{0}g_{\beta }+S_{\beta }\sigma_{\beta }.$$

In these equations, g_β_ (g_α_) represent the bulk Gibbs free energy per atom (the bulk energy density) of the β phase (α phase), while σ_β_ (σ_α_) represent the specific surface energy (energy per unit area) of the β phase (α phase). The surface areas of the particles are given by *S*_α_ = 4π*R*_α_^2^ and *S*_β_ = 4π*R*_β_^2^; they depend on the atomic densities of α phase and β phase, respectively.

In the following, the letters “α” and “β” indicate the corresponding phases. For simplicity and convenience, we initially focus on the α→β transition and write all equations accordingly. It is evident that for the β→α phase change, one may replace the subscript “α” with “β” and vice versa.

Then, Equation 1 for the α→β phase change may be rewritten as:


[4] $$\Delta G_{\text{p}}=G_{\beta }-G_{\alpha }=N_{0}\left(g_{\beta }-g_{\alpha }\right)+S_{\beta }\sigma_{\beta }-S_{\alpha }\sigma_{\alpha }.$$

Hereby, in accordance with Equation 1, Δ*G*_bulk_ = *N*_0_(*g*_β_ − *g*_α_) and Δ*G*_surf_ = *S*_β_σ_β_ − *S*_α_σ_α_.

### Energy change under irradiation

To describe the energy change under irradiation, we need to incorporate the effects of vacancy saturation caused by radiation-induced point defects. This can be achieved by modifying the expression for the bulk Gibbs free energy change to account for the additional energy associated with vacancy saturation. Let us introduce the change of the Gibbs free energy of a particle under irradiation, Δ*G*, and the Gibbs free energy for creating point defects in a material, Δ*G*_pd_.

The formation of defects alters the initial state and the final stage, resulting in an increase in the energy of the nanoparticle, (*G*_α_ + Δ*G*_pd_(α)) for the α-phase nanoparticle and (*G*_β_ +Δ*G*_pd_(β)) for the β-phase nanoparticle. We assume that vacancies are present in both the initial and secondary phases, which are expected to precipitate under irradiation.

The expressions for the energies (Equation 2 and Equation 3) can be rewritten as follows:




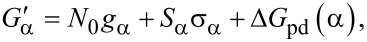







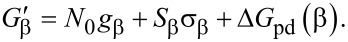




These equations represent the modified Gibbs free energies for the α-phase and β-phase nanoparticles, respectively, after considering the effects of defect formation. From this, the energy change, Δ*G*, for the α→β phase transformation under irradiation may be derived as:


[5] $$\begin{array}{c} \Delta G={G^{\prime }}_{\beta }-{G^{\prime }}_{\alpha }\\ =N_{0}\left(g_{\beta }\;-\;g_{\alpha }\right)\;+\;S_{\beta }\sigma_{\beta }\;-\;S_{\alpha }\sigma_{\alpha }\;+\;\Delta G_{\text{pd}}\text{​}\left(\beta \right)\;-\;\Delta G_{\text{pd}}\text{​}\left(\alpha \right). \end{array}$$

By introducing the notation, Δ*G*_pd_ = Δ*G*_pd_(β) − ΔG_pd_(α), the last expression can be rewritten as:


[6] $$\Delta G=\Delta G_{\text{bulk}}+\Delta G_{\text{surf}}+\Delta G_{\text{pd}}.$$

### Phase transformation criterion

Phase transition is thermodynamically possible only when the relationship for the change in Gibbs free energy is fulfilled:


[7] $$\Delta G_{\text{p}}<0\text{ for a nanoparticle without irradiation,}$$

[8] ΔG<0  for a nanoparticle under irradiation.

In the following, the condition Δ*G* < 0 for a nonzero particle diameter *d* (or *N*_0_) indicates the occurrence of the phase transformation and is used as the phase transformation criterion.

### Infinite case

Additionally, we investigate the behavior of the bulk under irradiation and the saturation of vacancies, assuming an infinite size where the surface terms are negligible. In this case, as *d*→∞ (or *N*_0_→∞), the surface terms can be neglected (|Δ*G*_bulk_| ≫ |Δ*G*_surf_|), and one can find the energy difference, Δ*G* ≈ Δ*G*_∞_, as the combination of the bulk thermodynamic stimulus and the energy for creating point defects Δ*G*_pd_:


[9] $$\begin{array}{c} \Delta G_{\infty }=\Delta G_{\text{bulk}}+\Delta G_{\text{pd}}\\ =N_{0}\left(g_{\beta }-g_{\alpha }\right)+\Delta G_{\text{pd}}\left(\beta \right)-\Delta G_{\text{pd}}\left(\alpha \right). \end{array}$$

The phase transition in an irradiated bulk material is thermodynamically favorable if the following condition is met:


[10] $$\Delta G_{\infty }<0.$$

### Energy of radiation-induced vacancies

The specific effects of irradiation on the energy change depend on the details of the material and the irradiation conditions and may require further analysis or experimental data for accurate characterization. In our case, the energies of point defects, Δ*G*_pd_(α) and Δ*G*_pd_(β), depend on the vacancy concentrations (denoted as *C*_v_^α^ and *C*_v_^β^, respectively) and can be expressed as follows [17]:


[11]

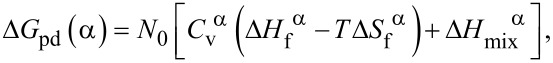




[12]

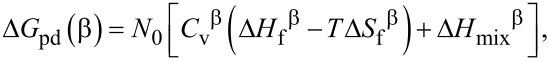



where, Δ*H*_f_ is the enthalpy change for forming of a vacancy, Δ*S*_f_ is the entropy change for vacancy formation, and Δ*H*_mix_ is the ideal entropy of vacancy mixing, which may be given as:


[13]

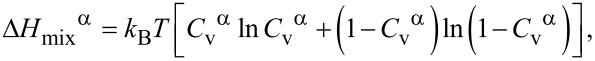




[14]

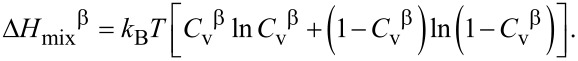



Here, *T* is the absolute temperature, and *k*_B_ is the Boltzmann constant.

### Chemical rate theory approach

The chemical rate theory approach involves the application of concepts from chemical kinetics to describe the evolution of defects in materials under irradiation. It considers the rates of defect formation, migration, and annihilation processes and aims to predict the steady-state concentrations of these defects under given irradiation conditions. In this approach, the rates of defect formation and annihilation are described by kinetic equations, which may be derived from fundamental principles such as the laws of thermodynamics and statistical mechanics. These equations typically involve parameters such as activation energies and defect concentrations, and they can be solved to obtain the steady-state concentrations of defects.

According to chemical rate theory, which incorporates the effect of particle interface sinks, steady-state concentrations of interstitials and vacancies in a material can be determined by considering two extreme cases, namely, (i) the case of vacancy–interstitial recombination, where defects are annihilated through recombination reactions, and (ii) the case of particle interface sinks, where defects are trapped and annihilated at external boundaries. These two cases represent different mechanisms for defect annihilation and can lead to different steady-state concentrations of defects depending on the material and irradiation conditions [17,21].

The time-dependence of the vacancy concentration, *C*_v_, and interstitial concentration *C*_i_ can be described by kinetic equations taking into account recombinations [22–24]:


[15] ∂Cv/∂t=Kv−ReCvCi−KdDvCv,

[16] ∂Ci/∂t=Kv−ReCvCi−KdDiCi.

Here, *K*_v_ is the defect generation rate (or atomic displacement rate, displacements per atom per second), Re is the recombination coefficient, *K*_d_ is the sink strength at the interface or external boundary (assumed equal for both vacancies and interstitials, *K*_d_ = 57.6/*d*^2^), and *D*_v_ and *D*_i_ are the diffusion coefficients for vacancies and interstitials, respectively.

In the following, we suggest that defect annihilation in HDCMs is dominated by nanoparticle surface sink effects, where interstitials rapidly migrate to the surface sink and recombine with vacancies located at the particle surface or interphase boundary. (The concentration of interstitials becomes much smaller than the vacancy concentration, while the diffusion coefficient of interstitials is much larger than the diffusion coefficient of vacancies). In this case, the point defects in HDCMs under irradiation are mainly vacancies inside the nanoparticle, and the movement of interstitials from their initial positions to the surfaces is assumed to be rapid [17,22–24]. Additionally, the nanoparticles are considered isolated, with no exchange of atoms between them, making the saturation of vacancies inside the nanoparticle the primary factor for irradiation effects in HDCMs. It is also assumed that the concentration of radiation-induced point defects at characteristic temperatures exceeds the concentration of thermal-equilibrium defects and that there are no other reservoirs besides the surface of the particle.

In the steady-state regime for small nanoparticles, recombinations are unimportant, and the time-dependence of the vacancy concentrations *C*_v_^α^ in the α phase and *C*_v_^β^ in the β phase can be described by kinetic equations [17,25]:


[17]

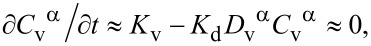




[18]

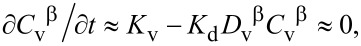



where, *D*_v_^α^ and *D*_v_^β^ are the diffusion coefficients for vacancies in the α phase and the β phase, respectively:


[19]

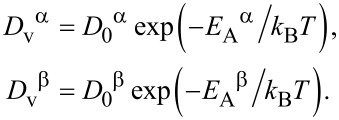



Here, *D*_0_^α^ and *D*_0_^β^ are the kinetic coefficients related to the jump frequencies of vacancies in α phase and β phase, respectively; *E*_A_^α^ and *E*_A_^β^ are the vacancy activation energies (usually specified through vacancy migration energies) in α phase and β phase, respectively. The diffusion coefficients for vacancies vary exponentially with the activation energies, *E*_A_^β^ and *E*_A_^α^, divided by temperature. *D*_0_^α^ and *D*_0_^β^ are determined by the number of neighboring atoms. The diffusion coefficients of vacancies can also be calculated using the self-diffusion coefficients in a monovacancy mechanism mediated by nearest-neighbor vacancy jumps [26–27].

It is important to note that Shen’s approach assumes a size-dependence of the vacancy concentration in the steady-state regime (Equations 15 and 16), that is, the concentrations are proportional to the square of the particle size, *R*^2^:


[20]

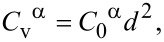




[21]

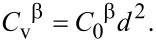



Here, *C*_0_^β^ = *K*_v_/(57.6*D*_v_^β^) and *C*_0_^α^ = *K*_v_/(57.6*D*_v_^α^) are the proportionality factors, and the vacancy diffusion coefficients determine the proportionality factors *C*_0_ in Equations 11–14. In the following, we employ the steady-state approach (Equations 17–21) in chemical rate theory.

For relatively small vacancy concentrations (that we usually deal with), the energies of point defects Δ*G*_pd_(α) and Δ*G*_pd_(β) increase almost linearly with *C*_v_^α^ and *C*_v_^β^. Hence, the size-dependent behavior of point defects leads to a size-dependent behavior of Δ*G*_pd_(α) and Δ*G*_pd_(β). In the steady-state regime, a nanoparticle with larger *R* should possess greater Δ*G*_pd_(α) and Δ*G*_pd_(β) because of the increased concentrations of radiation vacancies in the particle interior.

### Experimental study of point defects

The expressions in Equations 20 and 21 are justified in some experimental cases. Rose et al. used transmission electron microscopy to investigate nanoscale Pd under 240 keV Kr ion irradiation with a flux of 10^13^ ions·cm^−2^·s^−1^. Pd had grain sizes, *d*, ranging from 10 to 100 nm. The authors found that the defect density increases with increasing grain size *d*, and the slope between defect concentration and grain size is about 1.4, but not 2 as used in Shen’s model [3]. An experimental study on ZrO_2_ with grain sizes ranging from 10 to 300 nm showed that the number of defects per volume (defect clusters per cubic nanometer) can be fitted by an expression like Equation 13 with a fitted exponent of about 2 [18]. Wei et al. investigated typical bcc metals and found that (i) vacancy accumulation is lower in metals with small vacancy migration energies, such as V, Cr, Fe, and Nb, and (ii) the relationship between vacancy concentration and grain size (ranging from 10^−9^ to 10^−6^ m) under an irradiation rate of 10^−6^ dpa·s^−1^ at 600 K follows a power law with an exponent of about 2 (for different grain sizes from minimal to a critical value, after which the concentration becomes constant) [25].

In the following, we use quadratic dependences (Equations 20 and 21) for concentrations of defects.

### Probability aspect

The probability aspect is an important consideration when analyzing phase transitions. The probability of a phase transition to occur can be described by considering the relative stability of different phases and the energy barriers between them. In our case, the transformation of α phase to β phase is indeed a first-order phase transition, and Gibbsian thermodynamics can be used to estimate the probability and energies involved in the transformation. However, this approach does not directly provide information on the kinetics of how the transformation occurs. The probability of the phase transition (referred to as *p*) can be related to the energy difference between the initial and final states and can be described by an exponential function:




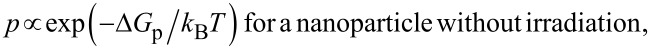







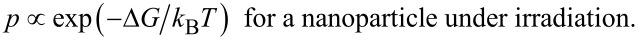




These equations illustrate that the probability of the phase transition decreases exponentially as the energy difference between the initial and final states increases. Therefore, phase transitions with higher energy barriers are less likely to occur at a given temperature. From this, one can consider the different configurations of α→β phase change and calculate the energies and the probabilities. Hence, one can gain insights into the thermodynamic feasibility and likelihood of the phase transition occurring under specific conditions.

For the graphical representation of the results, we introduce energy densities per atom as follows: Δ*g*_bulk_ = *g*_β_ − g_α_ = Δ*G*_bulk_/*N*_0_ represents the bulk Gibbs free energy change, Δ*g*_surf_ = Δ*G*_surf_/*N*_0_ represents the Gibbs free surface energy change, Δ*g*_p_ = Δ*G*_p_/*N*_0_ represents the total Gibbs free energy change of the particle without irradiation, Δ*g* = Δ*G*/*N*_0_ represents the total Gibbs free energy change of the particle under irradiation, and Δ*g*_pd_ = Δ*G*_pd_/*N*_0_ represents the energy change of defect formation. Our calculations demonstrate the importance of distinguishing between instability points (where the condition Δ*G*_p_ = 0 or Δ*G* = 0 is met) and phase stability zones (or radiation tolerance zones) in the temperature–size phase diagrams discussed here.

### Selection of the system and the type of transformation

Since the presented thermodynamic approach is general, we can consider various possible phase transformations under irradiation. For example, polymorphic transformation involving a change in lattice type and amorphization are two common types of transformations observed in metal nanosystems and ceramic substances, respectively. (As we know, most elements of the periodic table are metals.) Among the metals undergoing polymorphic transformations are Fe, Co, V, W, Ti, Tl, Zr, Sr, Mn, Al, Ga, Sc, Ba, Li, Na, and K. Therefore, it is important to examine a comprehensive range of quantities, including thermodynamic parameters, driving forces, and kinetic characteristics, under irradiation conditions. However, determining surface energies and activation energies of vacancy diffusion poses a particular challenge because of contradictory experimental data from various sources, which may lead to divergent results. Unfortunately, we were unable to find real systems with a complete set of the mentioned parameters. Therefore, we resorted to model approximations to qualitatively demonstrate possible situations.

However, during the search for systems and parameters, it became evident that substances with high activation energies of defect diffusion can serve as suitable candidates for radiation-induced phase transitions. Slow defect diffusion (Equation 19) results in high vacancy concentration (Equations 20 and 21), leading to a large energy change in defect formation (Equations 11 and 12). Therefore, it is important to consider the values of *E*_A_^α^ and *E*_A_^β^, typically specified in the case of irradiation through vacancy migration energies [17–18,20–27]:




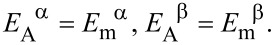




When comparing metals and ceramics, it becomes evident that ceramic substances typically exhibit high vacancy migration energy values. For instance, typical values for the migration energy of ceramics (*E*_m_^α^ and *E*_m_^β^) range from approximately 2.0 to 4.0 eV, while for metals, typical values (*E*_m_^α^ and *E*_m_^β^) range from 0.1 to 2.5 eV (osmium has *E*_m_ = 3 eV) [25–28]. As a result, ceramics should be better suited for describing radiation-induced phase transitions. Additionally, ceramics often amorphize rather than undergo polymorphic transformation. In such cases, the emerging amorphous phase is disordered and contains almost no radiation vacancies. Therefore, when considering the transition from the α phase to the amorphous phase (amorphization), it is necessary to exclude the concentration of vacancies in the β phase (*C*_v_^β^) and the energy contribution of vacancies in the β phase (Δ*G*_pd_(β) = 0).

#### Model of an iron-like nanomaterial with polymorphic phase transitions

As an example, in this work we consider a model of an iron-like nanomaterial with a polymorphic phase transition, for which we will subsequently generalize to other cases by varying parameters. Bulk thermodynamic data for pure iron have been sourced from various references to compile the set of parameters [29–41]. At low and intermediate temperatures, bulk Fe can exist in two crystallographic modifications, that is, the bcc phase (*T* < 1183 K) and the fcc phase (1183 K < *T* < 1665K). In this study, the bcc phase represents the model α phase, while the fcc phase represents the model β phase. Therefore, our focus will be on analyzing the transformations from bcc to fcc and from fcc to bcc that occur in an iron-like nanomaterial. We detail the findings for pure iron at the end of the paper.

The enthalpy change for vacancy formation can be estimated from the equilibrium melting temperature, *T*_m_, and is Δ*H*_f_^α^ = 3.76·10^−19^ J for the α phase and Δ*H*_f_^β^ = 3.28·10^−19^ J for the β phase [29–33]. The entropy change can be estimated using the Boltzmann constant, with values of Δ*S*_f_^α^ = −0.5*k*_B_ for the α phase and Δ*S*_f_^β^ = 0.2*k*_B_ for the β phase [29–34]. Regarding kinetic parameters, the diffusion coefficients are estimated as *D*_0_^α^ = 1.03·10^−3^ m^2^·s^−1^ and *D*_0_^β^ = 1.07·10^−3^ m^2^·s^−1^, while the vacancy migration energies are taken (in most cases as reference values) as *E*_m_^α^ = 4.96·10^−19^ J (3.1 eV) for the α phase and *E*_m_^β^ = 5.36·10^−19^ J (3.3 eV) for the β phase (close to ceramics) to demonstrate the vacancy effect clearly [17,34]. For comparison, in SiC, the energy of silicon vacancy migration is nearly 2.4 eV, and the energy of carbon vacancy migration is about 3.6 eV [42–44]. We focus on vacancy migration energies at the end of the paper and detail the findings. The surface energies σ_α_ and σ_β_ of an iron-like nanoparticle are estimated as 2.21 and 2.17 J·m^−2^, respectively, while the interphase energy is taken as σ_αβ_ = 0.04 J·m^−2^ according to data [38–41]. We focus on surface energies at the end of the paper and detail the findings for a pure iron nanoparticle. The volume density of atoms, *n*, varies within 1–2% (for example, at 1500 K, it is nearly 7.92·10^28^ m^−3^ for the β phase and 7.94·10^28^ m^−3^ for the α phase). Both the driving force of the transformation, Δ*g*_bulk_ = *g*_β_ − *g*_α_, and the density, *n*, are functions of the temperature [33,37–41,45]. The model parameters for irradiation include a defect generation rate *K*_v_ set at 10^−3^ to 10^−4^ dpa·s^−1^ [25].

## Results

### Model of the polymorphic α→β phase transition

#### Size effect and irradiation

**Figure 2:**
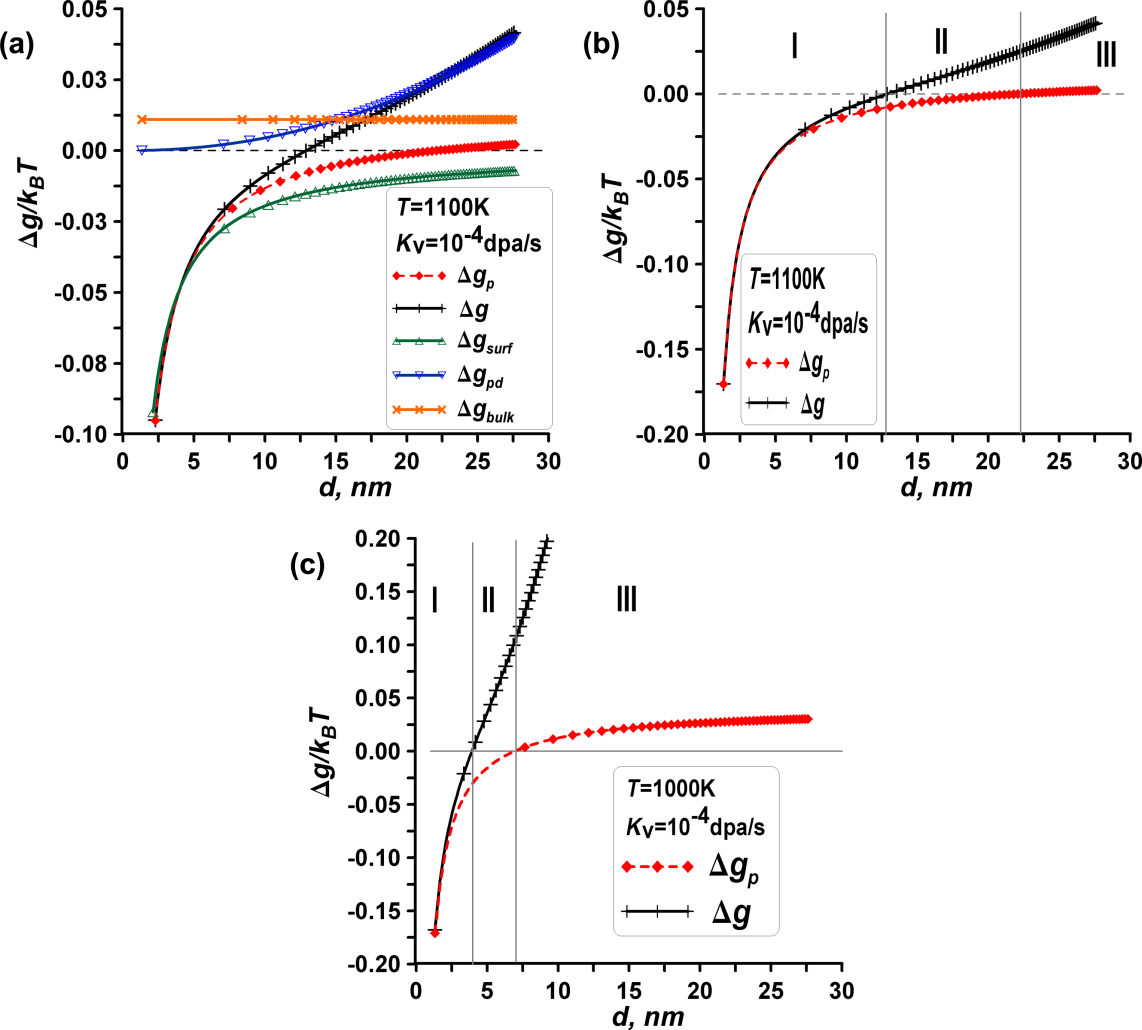
Comprehensive visualization of the energy changes for the model of an iron-like nanomaterial with polymorphic phase transitions. (a) Energy changes Δ*g* for the α→β phase transition, point defects, and surface energy as functions of size (*d*) and irradiation (*K*_v_). (b) Energy changes Δ*g* (represented by black crosses) and Δ*g*_p_ (by red points), illustrating the α→β phase transition in a spherical α-phase nanoparticle as a function of particle size, demonstrating three distinct zones, namely, zone I (Δ*g* < 0, Δ*g*_p_ < 0), intermediate zone II (Δ*g* > 0, Δ*g*_p_ < 0), and zone III (Δ*g* > 0, Δ*g*_p_ > 0). (c) Effect of low temperature on the shift of the intermediate zone II. Decreasing the temperature leads to the narrowing of zone II. At *T* = 900 K, zone II disappears.

Based on the provided information, we can apply the phase transformation criterion to a nanosized material, considering the energy change at a fixed temperature and under irradiation. The visualization in Figure 2 depicts the influence of irradiation on the phase transition, showing three distinct zones based on the energy changes, namely zone I without the manifestation of radiation effects and with the dominant influence of surface energies (Δ*g* < 0, Δ*g*_p_ < 0), zone II with manifestations of radiation effects (Δ*g* > 0, Δ*g*_p_ < 0), and zone III without the manifestation of radiation effects and with the dominant influence of bulk driving forces (Δ*g* > 0, Δ*g*_p_ > 0).

**Zone I – unstable α-phase particle**. Transformation can occur for α-phase nanoparticles (up to nearly *d*_1_ = 12 nm at *T* = 1100 K), regardless of whether the material is irradiated or not (indicating instability of the initial bcc phase). In this zone, the dominant mechanism is not radiation but rather surface effects associated with a decrease in surface energy during the phase change. Consequently, both functions Δ*g* and Δ*g*_p_ are negative in zone I.

**Zone III – stable α-phase particle**. Phase transformation cannot occur, either with or without irradiation, indicating the stability of the initial bcc phase due to the dominant influence of bulk driving force Δ*g*_bulk_. In this zone, both Δ*g* > 0 and Δ*g*_p_ > 0.

**Intermediate zone II – unstable α-phase particle without irradiation and stable α-phase particle under irradiation.** Phase transformation can occur without irradiation, resulting in the formation of the β phase and a decrease in surface energy. However, irradiation affects α phase and β phase differently. As a result, Δ*g* > 0, while Δ*g*_p_ < 0 in zone II. Consequently, irradiation increases the stability zone III for large α phase (bcc) particles and decreases the instability zone I for small α-phase particles towards smaller sizes, as indicated by the leftward shift in Figure 2b,c.

#### Nucleation energy criterion

The previous thermodynamic approach may be applied to various cases, such as size-dependent transitions. However, it only considers the initial and final single-phase stages of the transforming system. Another important aspect for nanoscale systems is nucleation, which involves the appearance and growth of a new β-phase nucleus, leading to the formation of a two-phase α+β system.

In the nanoscale case, the nucleation energy criterion is crucial for understanding phase transformations and the formation of new phases in materials. Nucleation represents a first-order phase transition and results in the creation of a new interphase surface, characterized by a corresponding specific interphase energy (σ_αβ_) and area (*S*_αβ_). Due to the interplay between bulk energy stimulus (Δ*G*_bulk_) and surface energy terms, the Gibbs free energy required to form a nucleus of a new phase reaches a maximum value, known as ΔG* (referred to as the nucleation barrier or the critical work of nucleus formation). It is important to note that the size of a nucleus is termed the critical size and may not necessarily coincide with the size of the entire particle denoted as *d*.

The nucleation energy criterion states that for nucleation to occur, the nucleation energy barrier must be surpassed. Referring to classic textbooks [46], one can formulate the nucleation energy criterion for phase formation as follows:


[22] $$\Delta G^{*}=50k_{\text{B}}T.$$

If the value of ΔG* is very high (greater than approximately 50*k*_B_*T*), then the phase transition is suppressed. Therefore, it is essential to consider nucleation and the nucleation barrier.

**Figure 3:**
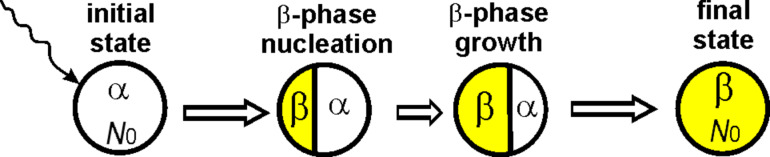
The cap-type (lenticular) nucleation mode for the α→β phase transition in a nanopowder under irradiation, showing the appearance of a new β-phase nucleus at the surface of an α-phase nanoparticle. The concentration of point defects in each phase varies with the size of the phase. The concentration in the β-phase nucleus changes with the volume of the β-phase nucleus, while the concentration in the remaining part of the particle also varies with changes in its volume.

To describe nucleation, it is necessary to consider the geometrical morphology of the transforming system and the possible transformation modes (Figure 3). In experiments, nucleation through a cap-type two-phase configuration has been observed, wherein a new surface-segregated phase grows in a layer-by-layer fashion, similar to epitaxial growth towards the inner region [47–50]. This type of growth is considered within the thermodynamic approach.

The next step is to develop a corresponding thermodynamic model. When the new β phase nucleates, the energy change must be determined for the two-phase α+β configurations shown in the middle of Figure 3. Let *N*_β_ and *N*_α_ = *N*_0_ − *N*_β_ be the numbers of atoms in β phase and α phase, respectively; σ_αβ_ and *S*_αβ_ are the specific interphase energy and area at the boundary of the β phase and the α phase, respectively.

Let us denote the change of the Gibbs free energy of the nanoparticle related to the formation of a new nucleus as Δ*G*_ncl_ and call it the nucleation energy. The nucleation energy is a function of the number of atoms in the new-phase nucleus Δ*G*_ncl_ ≡ Δ*G*(*N*_β_). The change of the Gibbs free energy Δ*G*_ncl_, corresponding to the formation of a new nucleus in the nanoparticle, can be expressed as:


[23] $$\begin{array}{c} \Delta G_{\text{ncl}}=N_{\beta }\left(g_{\beta }-g_{\alpha }\right)+{S^{\prime }}_{\beta }\sigma_{\beta }+S_{\alpha \beta }\sigma_{\alpha \beta }+\left({S^{\prime }}_{\alpha }-S_{\alpha }\right)\\ +\left[\Delta G_{\text{pd}}\left(\beta +\alpha \right)-\Delta G_{\text{pd}}\left(\alpha \right)\right]. \end{array}$$

The values *S*_α_, *S*_β_, *S*′_β_, *S*′_α_ and *S*_αβ_ represent the external surface areas of corresponding phases of the transforming α+β-phase particle and are assumed to be temperature-independent. To streamline the presentation and avoid the complexity of formula expressions accounting for nucleation morphology, we refrain from presenting the mathematical apparatus and detailed geometry descriptions. Similar analyses can be found in our previous work [45].

The energy of point defects, Δ*G*_pd_(β+α), can be expressed as follows:


[24]

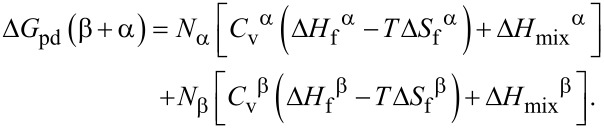



**Figure 4:**
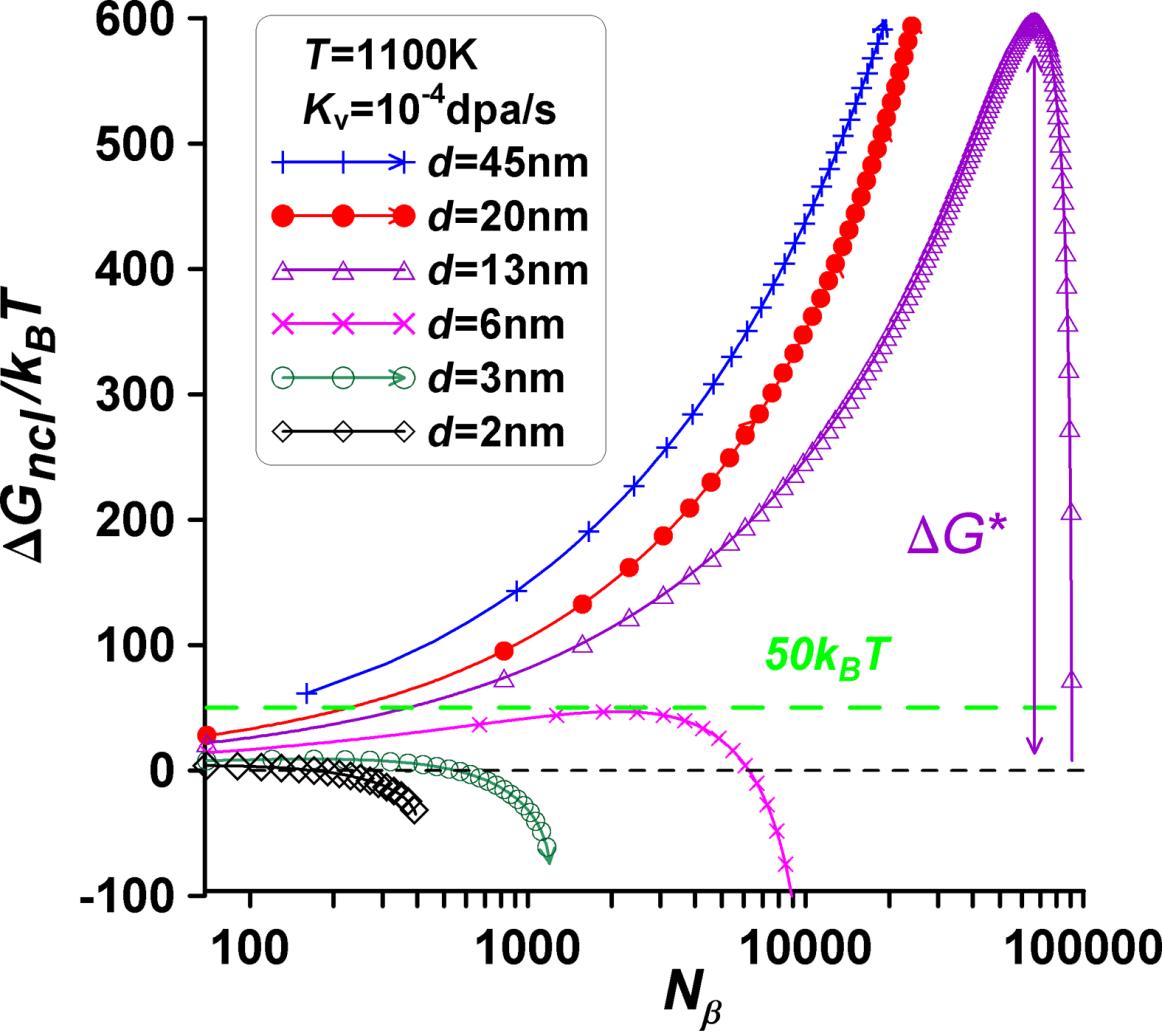
The nucleation energy change Δ*G*_ncl_ as a function of the number of atoms *N*_β_ at different fixed *N*_0_. The point *x* = 0 denotes the initial single α-phase particle, intermediate points describe cap-type two-phase α+β configurations, and the last points of all curves correspond to the single β-phase particle. The horizontal dotted line characterizes the nucleation energy criterion for phase formation Δ*G*_ncl_/*k*_B_*T* = 50.

Let us now visualize the dependence of Δ*G*_ncl_ ≡ Δ*G*(N_β_) on *N*_β_. The results of the computations for Equations 16 and 17 are shown in Figure 4 for different cases. The point *x* = 0 denotes the initial single α-phase particle, intermediate points describe cap-type two-phase α+β configurations, and the last points of all Δ*G*_ncl_ curves correspond to the single β-phase particle (final state on the right in Figure 4).

As one can see in Figure 4, nucleation in nanomaterials under irradiation treatment considerably changes the nucleation dynamics. Metastable two-phase configurations may exist due to the nucleation energy barrier, rather than stable ones. Phase transition can be prevented because of the very high value ΔG*. For instance, at a temperature of *T* = 1100 K, with *N*_0_ = 89000 and other parameters fixed, the transition from the α phase to the β phase is hindered by a high nucleation barrier. The nucleation barrier is about ΔG* = 575*k*_B_*Т* at the critical size of the new β-phase nucleus corresponding to *N*_β_ = 65000 (violet triangles in Figure 4). The concentration of vacancies in the bcc α phase is found as *C*_v_^α^ = *C*_v_^α^(*N*_α_) = 0.002%, the concentration of vacancies in the fcc β phase is *C*_v_^β^ = *C*_v_^β^(*N*_β_) = 0.05%.

#### Generalization of nucleation energy and phase transformation criteria

**Figure 5:**
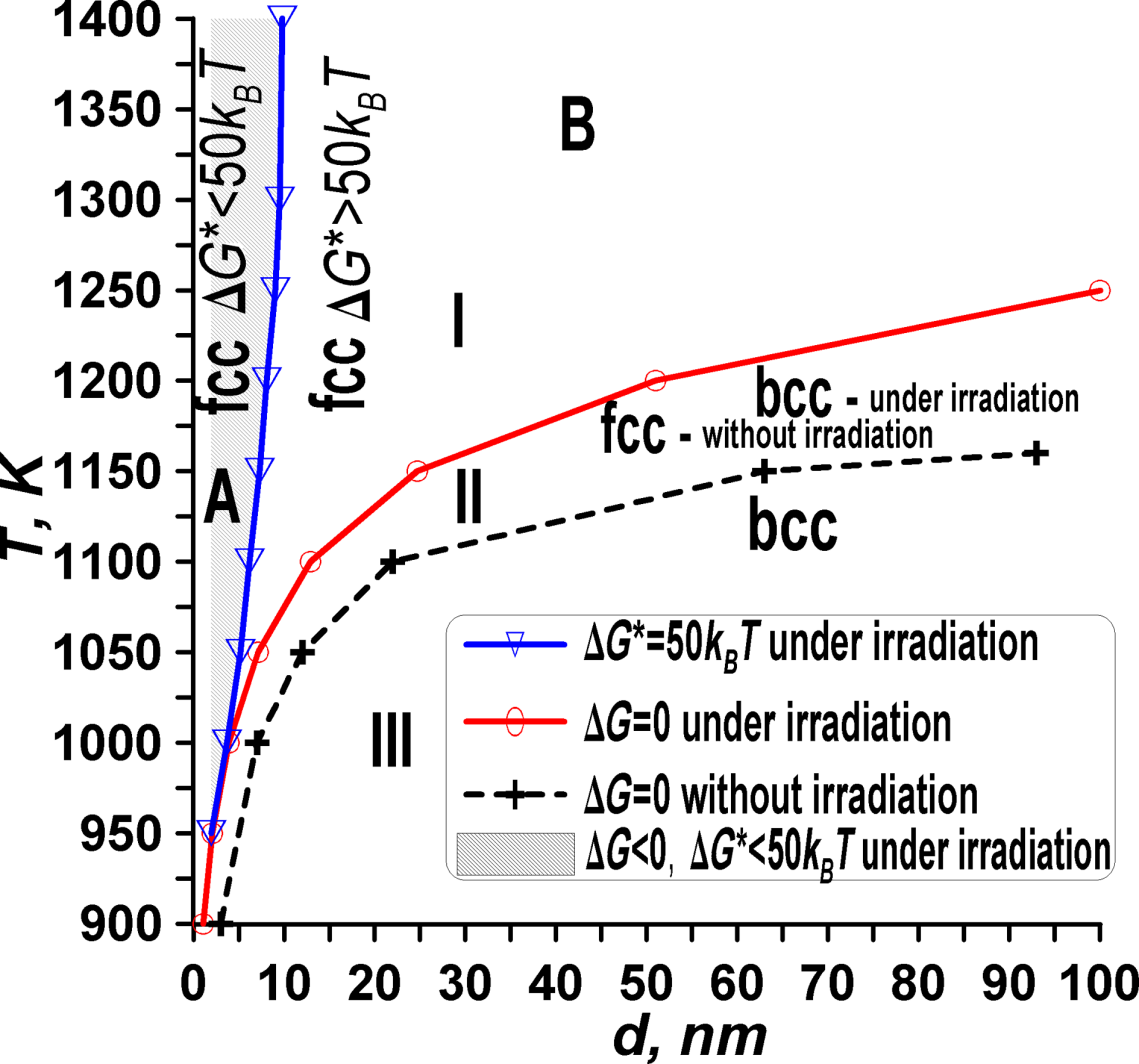
Size-dependent and temperature-dependent α-phase stability diagram for the model of an iron-like nanomaterial with polymorphic phase transitions under irradiation. The diagram outlines three distinct areas: area I of α-phase instability with *Δg* < 0, Δ*g*_p_ < 0, intermediate area II with Δ*g* > 0, Δ*g*_p_ < 0, and area III of α-phase stability with Δ*g* > 0, Δ*g*_p_ > 0. Area I is divided in two parts, that is, subarea A, characterized by a small nucleation barrier ΔG* < 50*k*_B_*Т* and subarea B, characterized by a high nucleation barrier ΔG* > 50*k*_B_*Т*.

The results can be generalized for different particle sizes, allowing for the creation of a size- and temperature-dependent diagram depicting the stability of the α phase. This diagram, referred to as the temperature–size α-phase stability diagram (Figure 5), illustrates three main areas (or four subareas) representing the transformation of α-phase nanoparticles under irradiation.

**Area I – unstable α-phase particle.** In this region, the phase transformation from the α-phase to another phase can occur, regardless of whether the material is irradiated or not, as indicated by Δ*g* < 0, Δ*g*_p_ < 0. Within area I, there are two distinct regions: In one part of area I (subarea A), the nucleation process requires only a small additional energy change, denoted as ΔG* < 50*k*_B_*Т*. Consequently, the probability of nucleation through cap-type two-phase configurations (see Figure 3) is high. In the other part of area I (subarea B), the nucleation process requires a large additional energy change, denoted as ΔG* > 50*k*_B_*Т*. Here, the probability of phase change fluctuations is small because of the high energy barrier.

**Intermediate area II – unstable α-phase particle without irradiation and stable α-phase particle under irradiation.** The intermediate area II represents an unstable α-phase particle without irradiation and a stable α-phase particle under irradiation. Within this region, the phase transformation from α-phase to another phase can occur through different mechanisms, characterized by Δ*g* > 0 and Δ*g*_p_ < 0. In this area, irradiation plays a crucial role in increasing the stability of the α phase (bcc). While without irradiation, the α-phase particle is unstable, the α phase becomes stable under irradiation. This increase in stability under irradiation expands the stable region for the α phase, leading to a larger area where the phase remains unaffected by external factors.

**Area III – stable α-phase particle.** In area III, the α-phase particle is stable, regardless of whether the material is irradiated or not. This stability is indicated by Δ*g* > 0 and Δ*g*_p_ > 0. Within this region, the phase transformation from the α phase to another phase cannot occur, as the free energy change (Δ*g*) is positive and the gradient of the free energy curve (Δ*g*_p_) is also positive. In other words, area III denotes a state where the α phase is thermodynamically favored and resistant to phase transformations. This stability holds true even in the presence of irradiation, indicating the robustness of the α phase under various external conditions.

It is worth noting that area I in Figure 5 corresponds to zone I in Figure 2, and similarly, area II in Figure 5 corresponds to zone II in Figure 2. This correspondence highlights the significant impact of nucleation on the phase transition dynamics. Specifically, the presence of nucleation fundamentally alters the situation, leading to the possibility of phase transition inhibition due to the existence of a very high energy barrier.

This observation underscores the critical role of nucleation phenomena in determining the feasibility and kinetics of phase transitions in materials. When nucleation is considered, it becomes apparent that the phase transition process may not always proceed as expected, especially in cases where the energy barrier for nucleation is prohibitively high.

### Reverse β→α polymorphic phase transition

Let us now consider the opposite situation where we examine the phase transformation from the β phase to the α phase (Figure 1b), starting with a β-phase nanoparticle (say, at high temperatures). We will repeat the analysis and rewrite Equations 1–24 by replacing the symbol “α” with “β” and vice versa. Performing calculations for the originally β-phase (fcc) particle will allow us to validate the results obtained for α-phase (bcc) particle and identify the characteristic features of nucleation and behavior under irradiation conditions. This case may also be considered more realistic in certain scenarios, particularly because during irradiation the newly formed phase typically has a lower atomic density. As mentioned, we will not present the corresponding formulas here to reduce the volume of the publication. However, conducting this analysis will provide valuable insights into the phase transformation dynamics from β phase to α phase, complementing the findings obtained for the case of the α phase particle.

#### Size effect and irradiation

**Figure 6:**
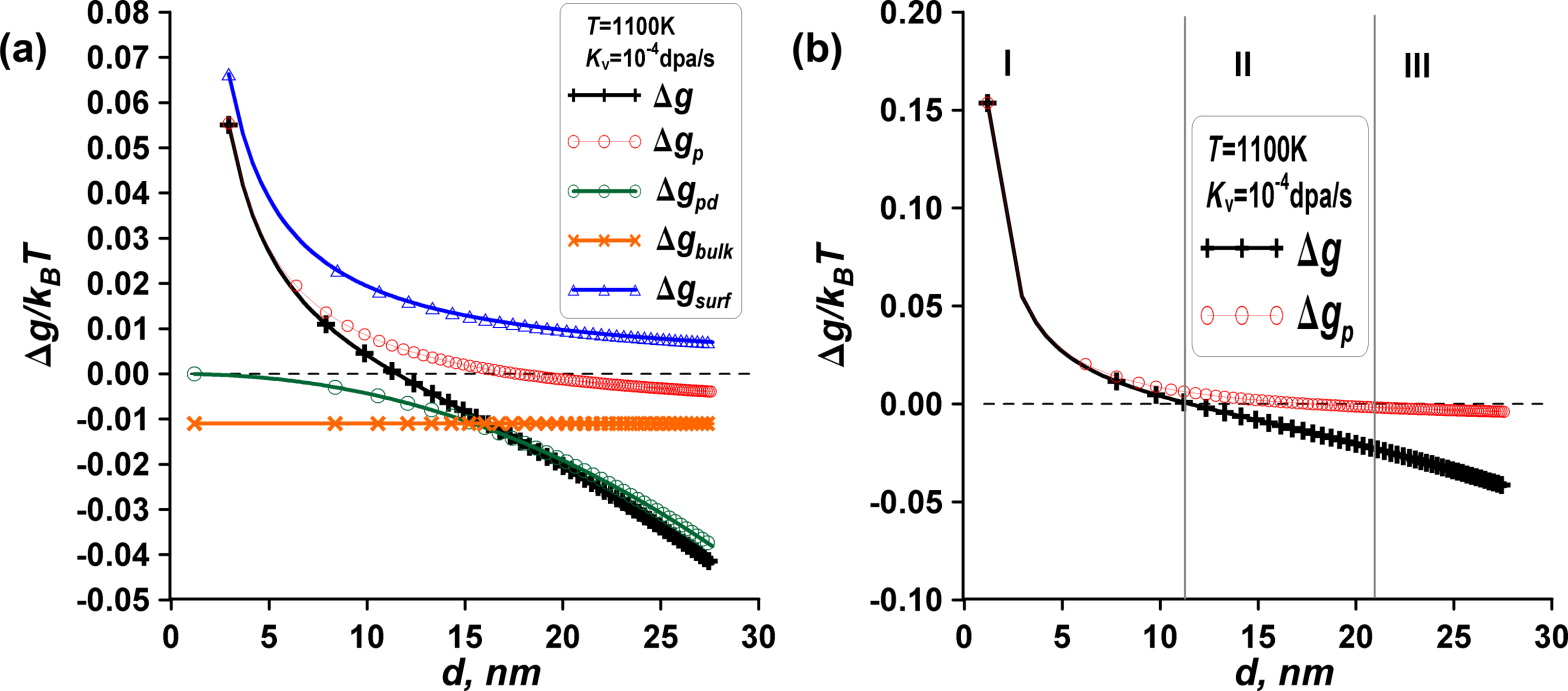
Comprehensive visualization of the energy changes. (a) Energy changes Δ*g* for the β→α phase transformation, for point defects, and for the surface energy as functions of the size *d* and the irradiation (*K*_v_). (b) Free energy changes for the β→α phase transformation as functions of the particle size *d* and illustrations of three zones: zone I of a stable β-phase particle (Δ*g* > 0, Δ*g*_p_ > 0), intermediate zone II (Δ*g* < 0, Δ*g*_p_ > 0), and zone III of an unstable β-phase particle (Δ*g* < 0, Δ*g*_p_ < 0).

We check the phase transformation criterion for a nanoscale material. Figure 6 shows a typical plot for energies at a fixed temperature of 1100 K and visualizes the energy change dependencies on size *d*. For the mentioned set of parameters, the instability point is found to be nearly *d*_1_ = 11.6 nm. At that point, Δ*g*_bulk_/*k*_B_*T* = −0.011, *N*_0_ = 66000, the bulk energy change is Δ*G*_bulk_/*k*_B_*T* = 730, the surface energy change is Δ*g*_surf_/*k*_B_*T* = 0.018 or Δ*G*_surf_/*k*_B_*T* = 1150, the energy of point defects is Δ*g*_pd_/*k*_B_*T* = −0.006 or Δ*G*_pd_/*k*_B_*T* = −400, and the vacancy concentration is *C*_v_^β^ = 0.00046.

Similar to the aforementioned case, we observe three zones on the energy change-size diagram for β-phase (fcc) nanoparticles. In zone I, corresponding to stable β-phase particles of small sizes, the transformation cannot occur as indicated by Δ*g* > 0 and Δ*g*_p_ > 0. In zone I, the influence of surface energies is dominant over other factors. In zone III, corresponding to unstable β-phase particles of large sizes, the β→α phase phase transition can occur with or without irradiation. In this phase, the fcc phase is unstable, while the bcc phase is stable, indicated by Δ*g* < 0 and Δ*g*_p_ < 0. In zone III, the influence of bulk driving forces is dominant over other factors. In the intermediate zone II, the phase transformation can occur under irradiation. Irradiation yields Δ*g* < 0, Δ*g*_p_ > 0 in zone II and decreases the width of stability zone I for small β-phase particles; also, it increases the width of unstability zone III for large β-phase particles towards smaller sizes. Decreasing the temperature leads to the narrowing of zone II. At *T* = 900 K zone II disappears.

Comparing our results to Shen’s work [17] on amorphization reveals significant differences in the number of phase stability regions. While we only identified three zones, Shen found five in the case of amorphization.

#### Nucleation energy criterion

**Figure 7:**
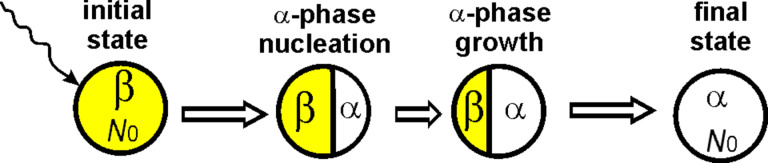
The cap-type (lentils) nucleation mode for the β→α phase transition in a nanoparticle under irradiation, depicting the emergence of a new α-phase nucleus at the surface of the β-phase nanoparticle. The concentration of point defects in each phase changes with the size of the phase.

In order to describe nucleation, it is essential to consider the geometrical morphology of the transforming system and the potential transformation modes (Figure 7).

**Figure 8:**
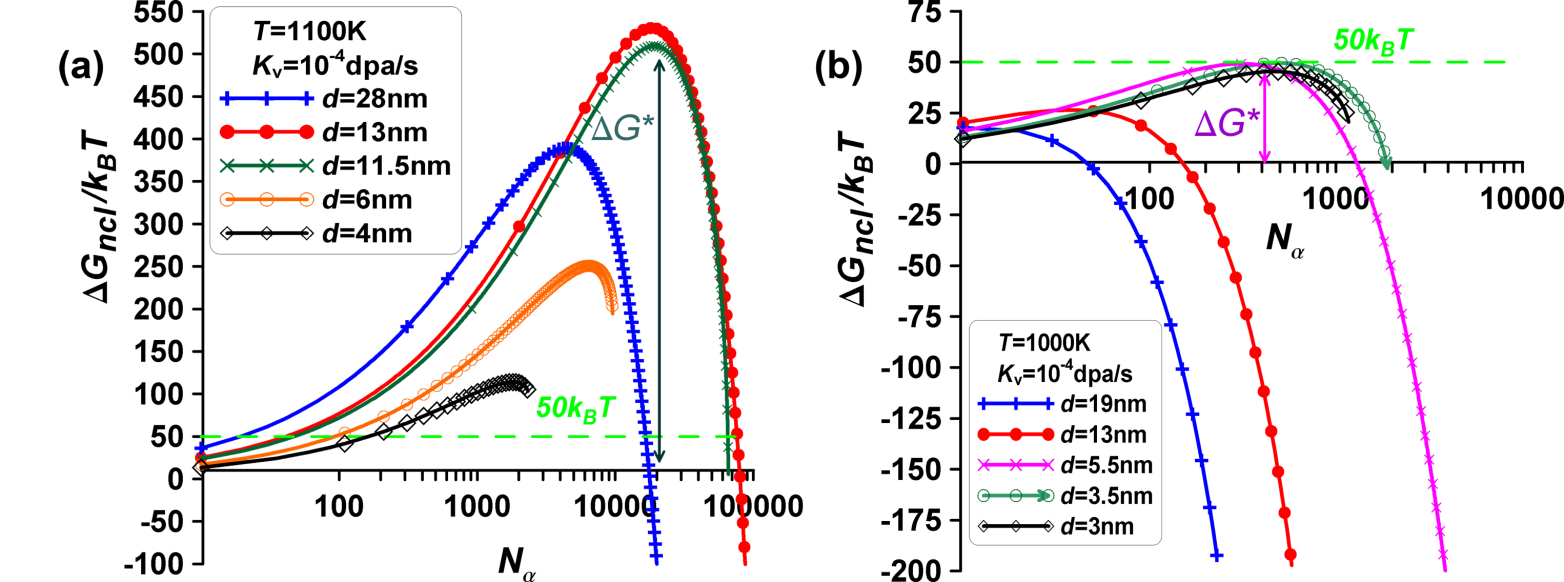
Energy difference Δ*G*_ncl_ as a function of the number of atoms *N*_α_ at different fixed sizes *d* (or *N*_0_) and temperatures, (a) *T* = 1100 K and (b) *T* = 1000 K. The points *x* = 0 represent the initial single β-phase particle, intermediate points depict cap-type two-phase α+β configurations, and the last points of all curves correspond to the final state of a single α-phase particle (refer to Figure 7). The horizontal dotted line represents the nucleation energy criterion for phase formation, Δ*G*_ncl_/*k*_B_*T* = 50. Metastable two-phase configurations are possible.

Let us now visualize the dependence of Δ*G*_ncl_ ≡ Δ*G*(*N*_α_) on *N*_α_. The results of the computations are depicted in Figure 8 for various cases. As shown in Figure 8, nucleation in nanomaterials under irradiation treatment significantly alters the situation. There is a possibility of metastable two-phase configurations instead of one stable phase due to the nucleation energy barrier. Phase transition can be inhibited because of the very high value of ΔG*.

At a temperature of *T* = 1000 K, with *N*_0_ = 6000 and other parameters fixed, the transition from the fcc β phase to the bcc α phase is hindered by a high nucleation barrier. The nucleation barrier is about ΔG* = 50*k*_B_*Т* at the critical size of the new α-phase nucleus corresponding to *N*_α_ = 450 (violet crosses in Figure 8b). Hereby, the concentration of vacancies in the α phase is found as *C*_v_^α^ = 0.003%, the concentration of vacancies in the β phase is *C*_v_^β^ = 0.3%.

#### Generalization of nucleation energy and phase transformation criteria

**Figure 9:**
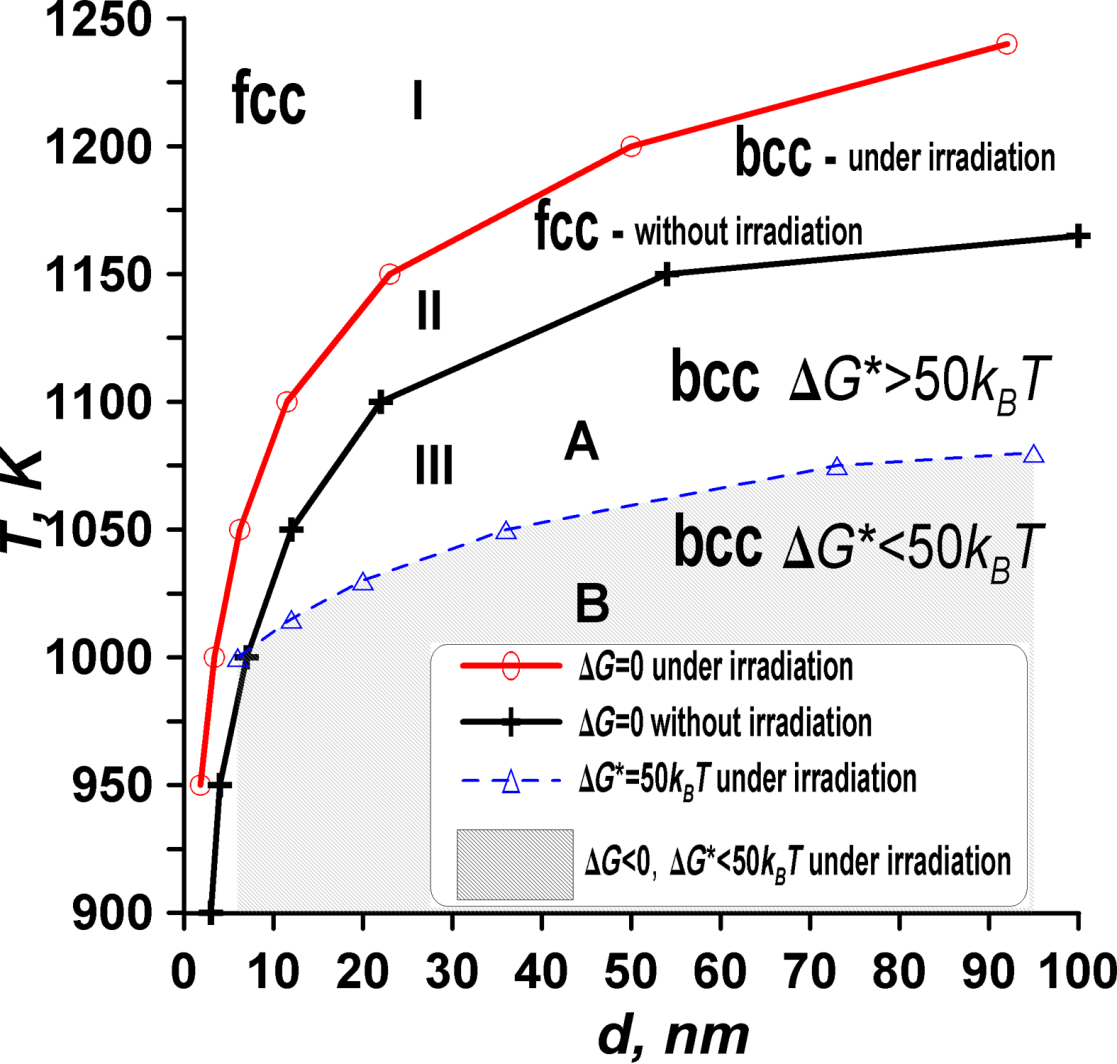
Size-dependent and temperature-dependent β-phase stability diagram for the β→α-phase transition under irradiation.

The results can be generalized for different particle sizes, allowing for the presentation of a size- and temperature-dependent diagram for the stability of the β phase (Figure 9). The diagram illustrates three areas (or four subareas) for the transformation of β-phase nanoparticles under irradiation.

**Area I – radiation tolerance and stability of the β-phase particle.** At high temperatures and small sizes, the phase transformation cannot occur, whether the material is irradiated or not. Even if the α phase is formed, it will remain metastable, indicated by Δ*g* > 0 and Δ*g*_p_ > 0.

**Intermediate area II – stable β-phase particle without irradiation and an unstable β-phase particle under irradiation.** The phase transformation can occur in different ways, as discussed above, indicated by Δ*g* < 0 and Δ*g*_p_ > 0.

**Area III – unstable β-phase particle.** For relatively large sizes and low temperatures, the phase transformation can occur regardless of whether the material is irradiated or not (Δ*g* < 0, Δ*g*_p_ < 0). In one part of area III (subarea A), nucleation requires a large additional energy change (ΔG* > 50*k*_B_*T*), and the probability of such phase change fluctuations is small. In another part of area III (subarea B), nucleation requires a small additional energy change (ΔG* < 50*k*_B_*T*).

## Discussion

### Effect of defect generation rate

**Figure 10:**
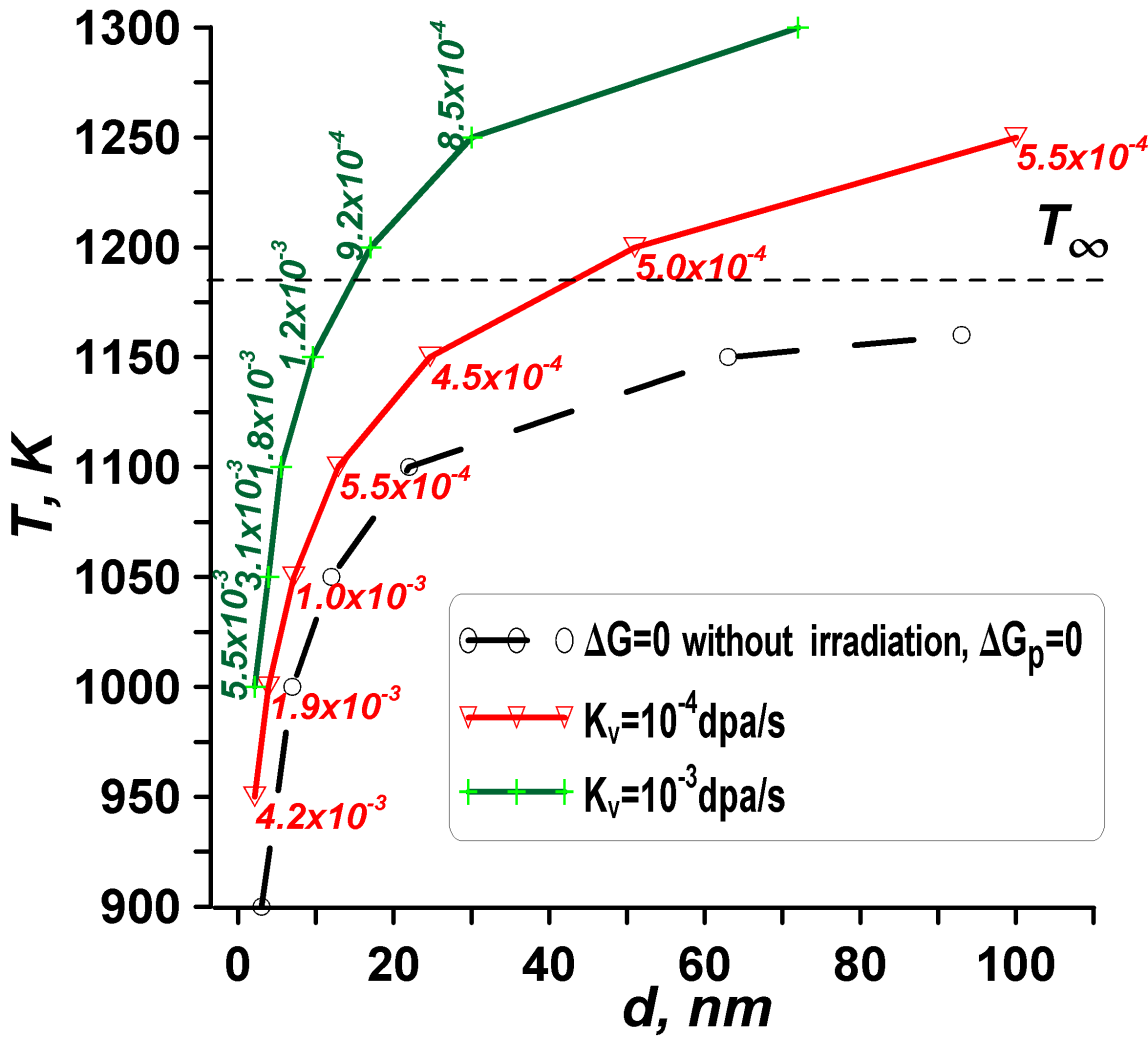
The impact of different defect generation rates on the shift of curves corresponding to the condition Δ*G* = 0 under irradiation (solid colour curves) in a size-dependent and temperature-dependent β-phase stability diagram. The dashed black curve represents the condition Δ*G*_p_ = 0 without irradiation. The numbers near the point symbols indicate the values of vacancy concentrations *C*_v_^β^ in the β phase.

At the conclusion of the description, we analyze the impact of defect generation rate (*K*_v_) on the behavior of the curves in the stability diagrams we obtained. We explore a case of the β phase transitioning to the α phase. Figure 10 presents size-dependent and temperature-dependent β-phase stability diagrams with varying defect generation rates *K*_v_ (atomic displacement rates). The numbers adjacent to the point symbols denote the values of vacancy concentrations *C*_v_^β^ in the β phase. *T*_∞_ = 1183 K is the transformation temperature for a bulk β-phase sample without irradiation.

As depicted in Figure 10, irradiation induces a displacement of the β→α phase transformation boundary curve towards higher temperatures and smaller sizes. Higher defect generation rates correlate with an expansion of the zone of phase stability for the α-phase (bcc) particles and an increase in radiation-induced concentrations.

The second observed result illustrated in Figure 10 concerns the saturation of vacancies within particles at constant defect generation rates *K*_v_. Notably, there is a non-monotonic behavior in the concentration of radiation-induced vacancies. Initially, for smaller particles and lower temperatures, the saturation of radiation-induced vacancies surpasses that of larger particles and higher temperatures. For example, at *K*_v_ = 10^−4^ dpa·s^−1^, calculations yield *C*_v_^β^ = 4.2·10^−3^ for *d*_1_ = 3 nm and *T* = 950 K, whereas, for *d*_1_ = 20 nm and *T* = 1150 K, *C*_v_^β^ = 4.8·10^−4^, indicating a decrease in saturation. However, for larger particles and higher temperatures, the vacancy saturation increases to *C*_v_^β^ = 5.5·10^−4^ for *d*_1_ = 100 nm and *T* = 1250 K.

### Vacancy saturation – comparision with the infinite case

Let us examine the β→α-phase transformation criterion for a bulk material under irradiation conditions (Equations 9 and 10). Again, the α phase exists at low temperatures, *T* < 1183 K. At a temperature *T* = 1250 K, and for the mentioned set of parameters (*K*_v_ = 10^−4^ dpa·s^−1^), the instability point is found as follows: radiation-induced concentration in the α phase (bcc) *C*_v_^α^ = 4·10^−5^, radiation-induced concentration in the β phase (fcc) *C*_v_^β^ = 4·10^−4^, when |Δ*g*_bulk_|/*k*_B_*T* = |Δ*g*_pd_|/*k*_B_*T* = 0.004 and Δ*G*_∞_ = 0.

In Figure 10, at a temperature of 1250 K, a concentration *C*_v_^β^ = 5.5·10^−4^ is observed for a nanoparticle size of about 100 nm. Remarkably, this value of *C*_v_^β^ is higher in the nanoparticle compared to the infinite case. Such a disparity indicates that the limit vacancy concentration (also termed vacancy saturation or concentration corresponding to the phase transition) for the phase transformation in the nanoparticle surpasses that in the infinite bulk sample. Understanding why and how vacancy saturation influences the phase stability of nanoparticles is essential; it is elucidated through energy changes as outlined in Equations 11 and 12. A comparison reveals that this phenomenon stems from the β phase exhibiting larger saturations than the α phase. For example, at a temperature of 1150 K, where the condition Δ*G* = G′_β_ − G′_α_ = 0 is met, we find *C*_v_^α^ = 3.5·10^−5^ and *C*_v_^β^ = 4.7·10^−4^. For a particle size of *d* = 22.7 nm, the energies of point defects are Δ*G*_pd_(α)/(*N*_0_*k*_B_*T*) = 3.9·10^−4^ and Δ*G*_pd_(β)/(*N*_0_*k*_B_*T*) = 4.8·10^−3^. At a temperature of 1100 K, the concentrations are *C*_v_^α^ = 4.2·10^−5^ and *C*_v_^β^ = 5.5·10^−4^ for *d* = 12 nm with energies Δ*G*_pd_(α)/(*N*_0_*k*_B_*T*) = 4.9∙10^−4^ and Δ*G*_pd_(β)/(*N*_0_*k*_B_*T*) = 6.6·10^−3^.

### Effect of vacancy migration energies

**Figure 11:**
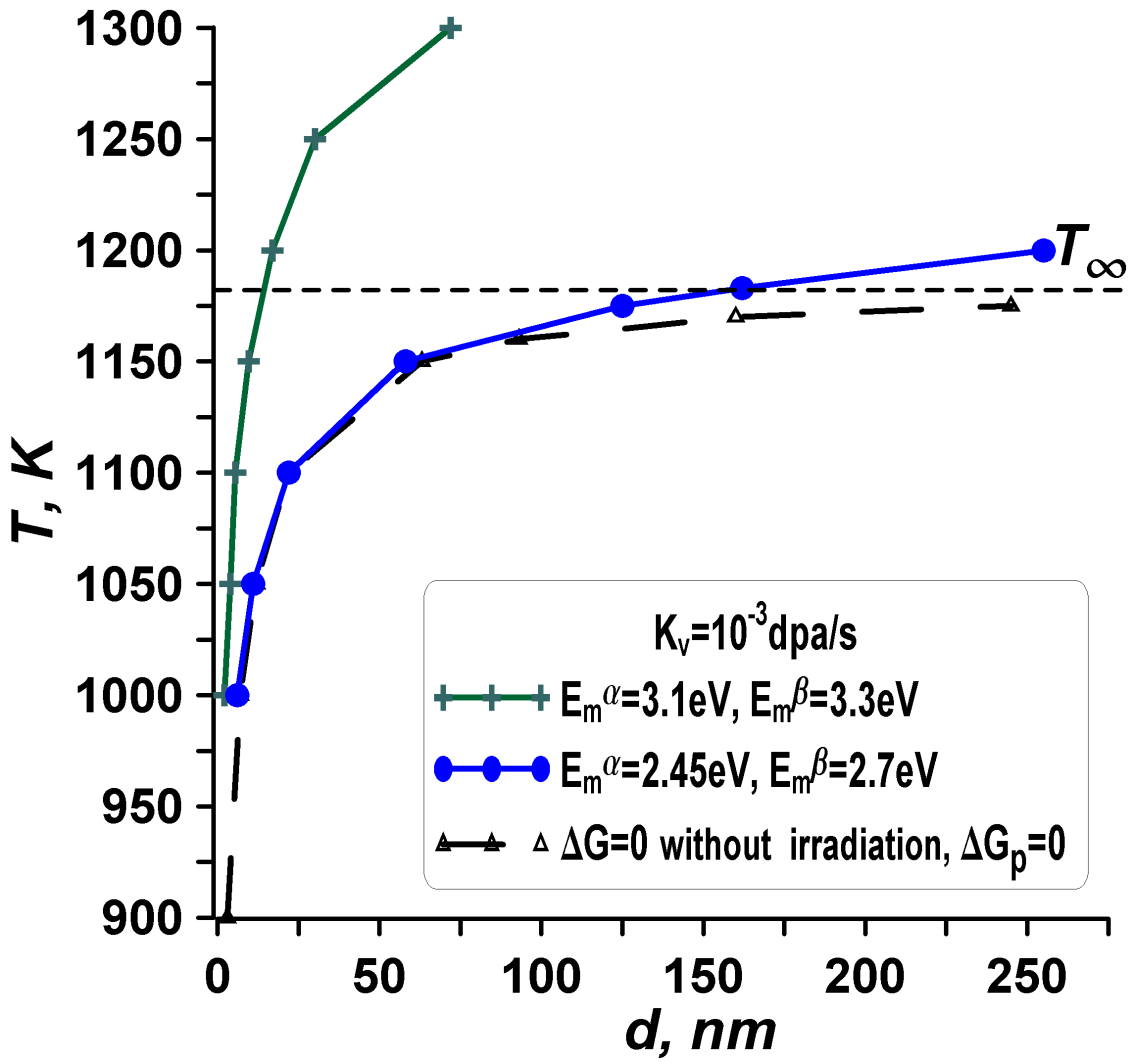
The impact of vacancy migration energies *E*_m_^α^ and *E*_m_^β^ on the shift of curves corresponding to the condition Δ*G* = 0 under irradiation within the phase stability diagram. The dashed black curve represents the condition Δ*G*_p_ = 0 without irradiation. A slight decrease in the migration energy of vacancies (by 20% of the considered model values) leads to a disappearance of the radiation effect.

Let us focus on the vacancy migration energies *E*_m_^α^ and *E*_m_^β^ to discern their influence on the radiation tolerance and phase stability of nanoparticles under irradiation. This understanding is facilitated by examining energy changes using Equations 11–14 and concentrations outlined in Equations 19–21. Figure 11 provides a visual representation of the stability diagram for different vacancy migration energies *E*_m_^α^ and *E*_m_^β^, while keeping the defect generation rate *K*_v_ and other parameters fixed.

Examining Figure 11 at a fixed temperature reveals that higher migration energies result in an expansion of the zone of phase stability for α-phase (bcc) particles and an increase in radiation-induced concentrations. Again, elevated energies of vacancy migration (*E*_m_^α^ and *E*_m_^β^) lead to smaller diffusion coefficients for vacancies (*D*_v_^α^ and *D*_v_^β^), resulting in greater concentrations (*C*_v_^α^ and *C*_v_^β^) and energy changes (Δ*G*_pd_(α) and Δ*G*_pd_(β)). A decrease in the migration energy of vacancies leads to an increase in the diffusion coefficients of vacancies, which leads to a decrease in the concentrations of radiation vacancies and a decrease in energy contributions (Δ*G*_pd_(α) and Δ*G*_pd_(β)). A slight decrease in the migration energy of vacancies (in the presented case by 20% of the considered model values) leads to a disappearance of the radiation effect. Such a decrease is equivalent to using metals instead of ceramics as material under consideration.

Comparing vacancy migration energies between metals and ceramics, we observe that crystalline ceramic substances typically exhibit significantly higher values. This suggests that the radiation-induced effect on phase changes in ceramics should be more pronounced. Conversely, in metals, the radiation effect on phase stability will be less noticeable, particularly in the crystalline state. Moreover, for normal radiation doses in the reactor core, a displacement rate *K*_v_ = 10^−8^ dpa·s^−1^ (10^−7^ dpa·s^−1^) corresponds to each atom being displaced once every three years (four months). Such reactor exposure may not induce significant phase changes in metals. Thus, under typical irradiation conditions and high temperatures, the low vacancy migration energy in metals results in such small defect concentrations that the contribution of irradiation energy to the total transformation energy of the particle is practically negligible (Equations 5 and 6).

In contrast, at low temperatures in small particles, the concentration of defects (vacancies) can increase sufficiently to initiate amorphization (or even premelting of the particle). We have already examined cases at high temperatures and observed that there is almost no radiation effect on metals. Therefore, it would be prudent to also consider the influence of irradiation and size on small particles at low temperatures.

### Small iron nanoparticles – effect of surface energies and low temperatures

At the end of our analysis, we focus on the case of very small iron particles ranging from 1 to 10 nm under irradiation at low temperatures. In this scenario, the irradiation doses are sufficient to establish a quasi-stationary regime for the appearance and annihilation of point defects. For example, considering 5 nm iron particles exposed to irradiation doses of approximately 0.1 dpa and displacement rates of *K*_v_ = 2·10^−3^ dpa·s^−1^, we can estimate a time of around 50 s required to reach a stationary state based on Equations 15 and 16. In this case, in a quasi-stationary regime, the primary point defects observed are vacancies in the γ-Fe (fcc) phase.

In the following, we use the vacancy migraton energy of γ-Fe *E*_m_(γ-Fe) = 1.4 eV for the non-magnetic fcc state (and *E*_m_ = 0.7 eV for the magnetic fcc state), according to [29–33]. For small iron particles, the question of how surface energies affect phase transformations in a nanoparticle under irradiation becomes relevant. It is also intriguing to explore how the effects of surfaces and radiation-induced defects can be decoupled when both influence phase transformations in the nanoparticle system concurrently. To address these questions, we consider two different cases of surface energies from various sources in the literature and compare the results of our calculations within the framework of the traditional thermodynamic approach, without accounting for nucleation.

**Figure 12:**
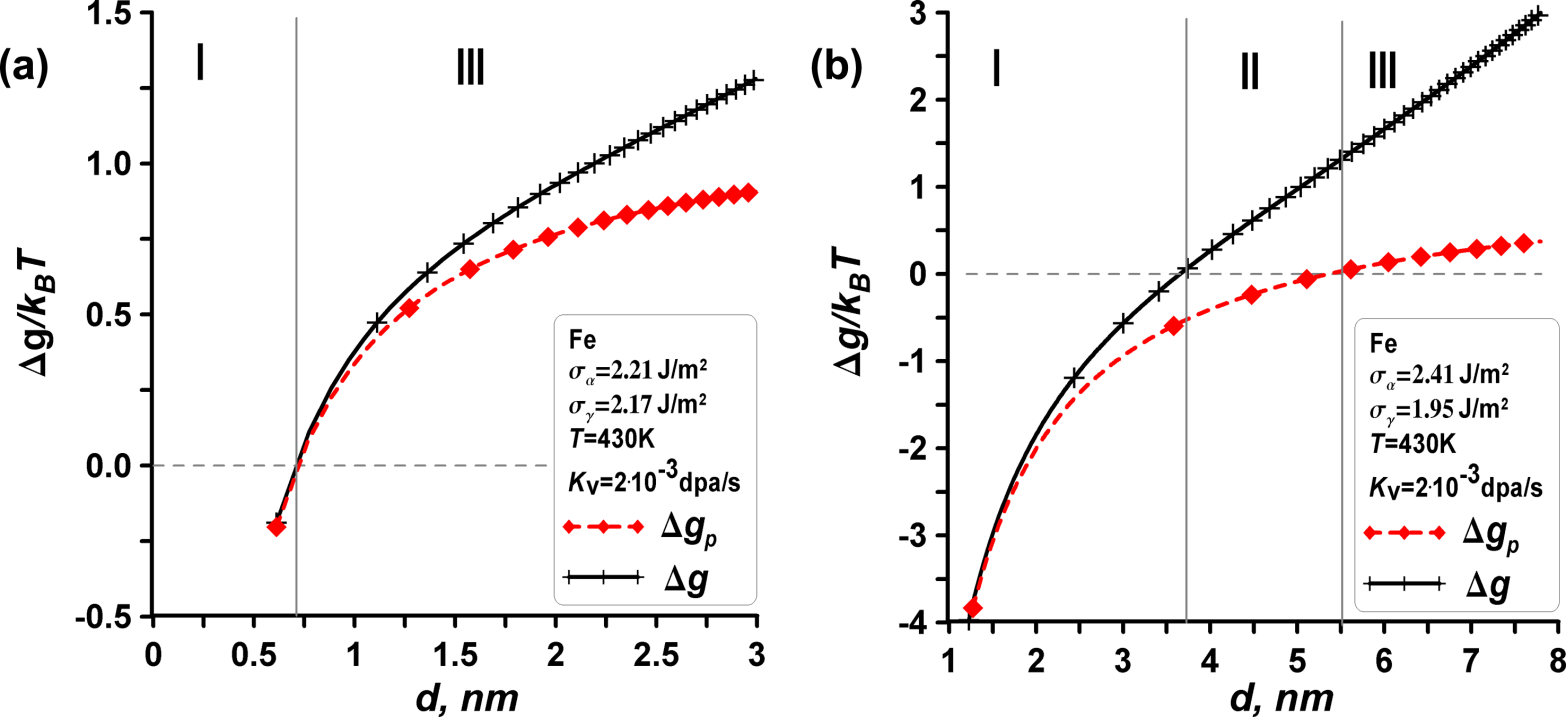
The impact of surface energies σ_α_ and σ_γ_ on the phase stability of small Fe nanoparticles. (a) First set of parameters according to [33,37,41] and (b) second set of parameters according to [51–52].

The first set of parameters (case (a)) for iron is chosen as follows: surface energy of the α-Fe bcc phase σ_α_ = 2.21 J·m^−2^ and surface energy of the γ-Fe fcc phase σ_γ_ = 2.17 J·m^−2^, according to [33,37–41]. The second set of parameters (case (b)) for iron is chosen as follows: surface energy of the α-Fe bcc phase σ_α_ = 2.45 J·m^−2^ and surface energy of the γ-Fe fcc phase σ_γ_ = 1.95 J·m^−2^, according to [51–52]. Using these two sets of parameters, we demonstrate that the ambiguity in experimental data on surface energies can significantly alter the results of calculations, potentially neutralizing interesting dimensional and radiation effects, as shown in Figure 12. Figure 12 elaborates on the general considerations presented in Figure 2 for the case of iron. From the comparison of Figure 12a and Figure 12b, it is evident that the choice of parameters is critical. In case (a), the intermediate zone II is virtually undetectable, whereas in case (b), zone II can extend to several nanometers.

Let us assume that the parameters are valid and examine case (b) in Figure 12b in more detail, focusing on the effects of radiation. Zone I represents Δ*g* < 0, Δ*g*_p_ < 0, zone II represents Δ*g* > 0, Δ*g*_p_ < 0, and zone III represents Δ*g* > 0, Δ*g*_p_ > 0. In zone I, the influence of surface energies predominates. The surface energy of the γ-Fe phase is lower than that of the α-Fe phase, rendering the γ-Fe phase stable, while the α-Fe phase is unstable. There is no radiation impact in zone I. In zone III, radiation effects (specifically the presence of radiation-induced vacancies in the phases) become also negligible compared to the bulk driving forces, indicating that a phase transition cannot occur, whether under irradiation or not, confirming the stability of the initial α-Fe phase. In Figure 12b, zone II is present and demonstrates the effects of radiation, indicating that radiation impacts phase transformations in nanoscale iron, but only within a very narrow range of sizes. For example, at *T* = 430 K, zone II appears to be between *d*_1_ = 3.75 nm and *d*_2_ = 5.5 nm. The concentration of vacancies in the γ-Fe phase is *C*_v_^γ^ = 1.1%, while the α-Fe phase has no radiation-induced point defects (*C*_v_^α^ = 10^−8^). The increased vacancy concentration in the γ-Fe phase aligns with findings reported in another study [53]. Comparing the Δ*g* and Δ*g*_p_ curves in Figure 12b, we can conclude that irradiation reduces the instability zone I for small α-Fe nanoparticles while expanding the stability zone III for larger α-phase particles, resulting in an intermediate zone II for stable α-Fe nanoparticles under irradiation. Additionally, zone II is also narrow in temperature. At *T* = 480 K, zone II disappears, leading to a behavior similar to that shown in Figure 12a. Here, the Δ*g* and Δ*g*_p_ curves almost coincide, since the vacancy concentrations decrease significantly with increasing temperature. At *T* = 400 K, in the quasi-stationary regime, the concentration of vacancies in α-Fe particles becomes negligible, while in the γ-Fe phase it increases significantly, from *C*_v_^γ^ = 5% for *d*_1_ = 2 nm to *C*_v_^γ^ = 35% for *d*_2_ = 5 nm. As a result, at low temperatures, the presented approach becomes inapplicable.

It is worth noting that spectroscopy, which provides vacancy formation enthalpies, and differential dilatometry, which determines equilibrium vacancy concentrations, indicate that the equilibrium vacancy concentrations in metals before the onset of melting range from 10^−4^ to 10^−3^ [10–12,54]. Hence, if abovementioned case (b) occurs, we can assume that iron particles might not only undergo polymorphic transformation due to irradiation and saturation with vacancies, but also melting. This issue is beyond the scope of the current study and will be addressed in future work.

### Remarks

The question regarding the fundamentality of the obtained results warrants careful consideration. It is notable that the model could be enhanced by incorporating dimensional dependencies of vacancy formation energy, surface energies, and diffusion coefficients. However, it remains uncertain whether the concentration or surface energy will increase or decrease. This challenge arises from the complexity of studying vacancy properties in solids, which involves numerous contradictory facts, primarily due to the difficulty of their direct observation.

Research has shown that vacancy saturation in free-standing nanomaterials, without irradiation and considered thermal equilibrium, can increase with size reduction and temperature rise because of the linear relationship between melting temperature and vacancy formation energy [55]. Since the melting temperature typically decreases for small particles [56], the vacancy formation energy may also decrease. Alternatively, the cohesive energy of solids can be considered proportional to the melting temperature [57–58], suggesting a correlation between the cohesive energy of solids and thermal vacancy formation energy.

Monte Carlo simulations using the broken bond model and molecular statics calculations with embedded atom method potentials show an opposite behavior, with the thermal vacancy concentration in metallic nanoparticles becoming smaller than the bulk value [59]. Furthermore, in embedded nanomaterials, the change in vacancy concentration may also differ [60]. Molecular dynamics simulations for the Fe system show that the melting temperature of bcc Fe only slightly reduces according to the vacancy concentration change [61].

## Conclusion

The present study aims to explore the influence of vacancy saturation and particle size on the phase transition of α-phase and β-phase nanoparticles under irradiation. This research highlights the potential for radiation-induced polymorphic transitions and delineates the zones of radiation stability for α-phase and β-phase nanoparticles. The primary factors contributing to the unique behavior of irradiated nanocrystalline α and β phases are the competition among the energy of accumulated vacancies within the nanoparticle, the bulk energy associated with phase transformation, the surface energy of the particle, and the steady-state approach in chemical rate theory to account for vacancy concentrations.

Three distinct zones are discerned in the energy change–size diagram. Nucleation considerations notably reshape the landscape, introducing the possibility of metastable two-phase configurations and hindering phase transitions due to high energy barriers.

For nanoparticles initially in the α phase that undergo α→β-phase transition, the following observations are made: In zone I, characterized by very small α-phase nanoparticles, the α→β phase transformation can occur regardless of irradiation (the bcc phase is unstable). The dominant mechanism in this scenario is associated with surface effects, which lead to a decrease in surface energy during the transformation. In zone III of large nanoparticles, the phase transformation cannot occur, regardless of irradiation (the bcc phase is stable). In the intermediate zone II, the α→β-phase change can occur without irradiation, and irradiation increases the stability zone III for large α-phase (bcc) particles and decreases the instability zone I for small α-phase particles.

We validated our results by conducting calculations for initially β-phase particles, yielding similar outcomes and revealing three distinct zones. Zone I indicates radiation tolerance and stability of β-phase nanoparticles; phase transformation cannot occur regardless of irradiation. In the intermediate zone II, the β-phase particle is stable without irradiation but becomes unstable under irradiation. In zone III, the β-phase particle is unstable, and the phase transformation can occur regardless of irradiation. In this zone, nucleation may necessitate a large additional energy change (>50*k*_B_*T*), resulting in a low probability of α→β phase change fluctuations.

Vacancy saturation significantly impacts the phase stability of model nanoparticles. This is primarily because the β phase exhibits larger saturations, nearly by one order of magnitude, than the α phase. The influence of radiation-induced vacancies leads to the expansion of the zone of phase stability of the α phase. Consequently, the limiting vacancy saturation of the phase transformation in the nanoparticle surpasses that in the infinite sample.

A comparison of the presented results with the conclusions drawn for amorphization, as demonstrated by Shen in [17], reveals discrepancies in the number of phase stability and instability regions. While this study for an iron-like nanomaterial with polymorphic phase transitions indicates three zones of phase stability resulting from the appearance of a new phase, amorphization may involve five regions. Additionally, there is a notable difference in the dependence of the Gibbs free energy change on nanoparticle size, which exhibits a non-monotonic behavior in the case of radiation-induced phase transformation of nanoparticles.

In the context of generalization, three main areas and four sub-areas are observed in the temperature–size phase stability diagram: (i) an area where the initial phase is stable and phase transformation is prohibited, (ii) an area where the initial phase is unstable, but the transition may be inhibited because of a high nucleation barrier or may occur easily because of a small nucleation barrier, and (iii) an intermediate area where the initial phase is unstable without irradiation but becomes stable under irradiation, or vice versa.

In the context of findings and generalization of the model, a radiation-induced transition from bcc to fcc is possible and depends on numerous parameters. The most promising candidates for radiation-induced effects are those with relatively low temperatures of phase transition and high vacancy migration energies. Furthermore, increasing the irradiation dose can enhance this effect. This precisely explains the results obtained for gold [16].

As a special case, we examined the phase stability of very small iron particles at low temperatures and sizes ranging from 1 to 10 nm. The results depend significantly on the chosen parameters and, unfortunately, cannot be deemed fully reliable and valid. However, if we accept the parameters for case (b) as valid and assume that the concentration of defects varies with size, we can demonstrate the potential influence of irradiation at low temperatures. Radiation narrows the stability zone I of the γ-Fe nanophase while expanding the stability zone III of the α-Fe nanophase towards smaller sizes.

In the steady-state regime, as particle sizes increase, the contribution of point defects also rises. However, for large metallic nanoparticles with sizes up to 200 nm, recombinations come into play, stabilizing the concentrations of point defects. As a result, point defects alone cannot account for phase transitions.

Comparing the vacancy migration energies for metals and ceramics reveals that the radiation effect on phase stability is generally less noticeable in bcc and fcc metals, and for reactor irradiation doses, it may not occur at all. Under ordinary irradiation conditions, the contribution of irradiation energy to the total energy of the nanoparticle is negligible.

Typically, the higher is the melting temperature, *T*_m_, the higher the vacancy migration energy, *E*_m_. For example, refractory metals include W with *E*_m_ = 1.7 eV and Ir with *E*_m_ = 1.63 eV. Most nanoscale metals exhibit superior resistance to irradiation, except for osmium, which has a relatively high vacancy migration energy of 3 eV. These metals may be recommended for nuclear materials. To observe radiation effects (involving point defects and vacancies) in phase transformations in most metallic particles, the irradiation dose needs to be increased by orders of magnitude.

## Data Availability

All data that supports the findings of this study is available in the published article and/or the supporting information to this article.
